# Zika Virus Infection Promotes Local Inflammation, Cell Adhesion Molecule Upregulation, and Leukocyte Recruitment at the Blood-Brain Barrier

**DOI:** 10.1128/mBio.01183-20

**Published:** 2020-08-04

**Authors:** Marion Clé, Caroline Desmetz, Jonathan Barthelemy, Marie-France Martin, Orianne Constant, Ghizlane Maarifi, Vincent Foulongne, Karine Bolloré, Yaël Glasson, Frédéric De Bock, Marine Blaquiere, Lucie Dehouck, Nelly Pirot, Edouard Tuaillon, Sébastien Nisole, Fatiha Najioullah, Philippe Van de Perre, André Cabié, Nicola Marchi, Fabien Gosselet, Yannick Simonin, Sara Salinas

**Affiliations:** a Pathogenesis and Control of Chronic Infections, INSERM, Université de Montpellier, Etablissement Français du Sang, Montpellier, France; b BioCommunication en CardioMétabolique, Université de Montpellier, Montpellier, France; c Institut de Recherche en Infectiologie de Montpellier, CNRS, Université de Montpellier, Montpellier, France; d Pathogenesis and Control of Chronic Infections, INSERM, Université de Montpellier, Etablissement Français du Sang, CHU Montpellier, Montpellier, France; e Réseau d'Histologie Expérimentale de Montpellier, BioCampus, CNRS, INSERM, Université de Montpellier, Montpellier, France; f Cerebrovascular Mechanisms of Brain Disorders, Institute of Functional Genomics, CNRS, INSERM, University of Montpellier, Montpellier, France; g Laboratoire de la Barrière Hémato-Encéphalique, Université d’Artois, Lens, France; h EA7524, Tropical and Infectious Disease Service, University of the Antilles, INSERM, Centre Hospitalier Universitaire de Martinique, Hôpital Pierre-Zobda-Quitman, Martinique, France; Washington University School of Medicine

**Keywords:** Zika virus, blood-brain barrier, cell adhesion molecules, leukocyte recruitment

## Abstract

The blood-brain barrier (BBB) largely prevents toxins and pathogens from accessing the brain. Some viruses have the ability to cross this barrier and replicate in the central nervous system (CNS). Zika virus (ZIKV) was responsible in 2015 to 2016 for a major epidemic in South America and was associated in some cases with neurological impairments. Here, we characterized some of the mechanisms behind its neuroinvasion using an innovative *in vitro* human BBB model. ZIKV efficiently replicated, was released on the BBB parenchyma side, and triggered subtle modulation of BBB integrity as well as an upregulation of inflammatory and cell adhesion molecules (CAMs), which in turn favored leukocyte recruitment. Finally, we showed that ZIKV-infected mouse models displayed similar CAM upregulation and that soluble CAMs were increased in plasma samples from ZIKV-infected patients. Our observations suggest a complex interplay between ZIKV and the BBB, which may trigger local inflammation, leukocyte recruitment, and possible cerebral vasculature impairment.

## Abstract (precis)

Zika virus (ZIKV) can be associated with neurological impairment in children and adults. To reach the central nervous system, viruses have to cross the blood-brain barrier (BBB), a multicellular system allowing a tight separation between the bloodstream and the brain. Here, we show that ZIKV infects cells of the BBB and triggers a subtle change in its permeability. Moreover, ZIKV infection leads to the production of inflammatory molecules known to modulate BBB integrity and participate in immune cell attraction. The virus also led to the upregulation of cellular adhesion molecules (CAMs), which in turn favored immune cell binding to the BBB and potentially increased infiltration into the brain. These results were also observed in a mouse model of ZIKV infection. Furthermore, plasma samples from ZIKV-infected patients displayed an increase in CAMs, suggesting that this mechanism could be involved in neuroinflammation triggered by ZIKV.

## INTRODUCTION

The central nervous system (CNS) is often considered an immune-privileged organ due to its separation from the bloodstream by various barriers, including the blood-brain barrier (BBB) and the blood-cerebral spinal fluid barrier (1). These barriers nonetheless allow selective passage of molecules and, especially, cells of the immune system (1, 2). The BBB represents a complex multicellular system that is tightly regulated: brain endothelial cells (BECs) form a tight endothelium, which is stabilized and dependent on the interaction with astrocytes, pericytes, and neurons, forming what is defined as the neurovascular unit (NVU) (2). Neurotropic viruses have been selected throughout evolution for their ability to access the CNS where, depending on their replication cycle and cellular effects, they cause a wide range of dysfunctions (3). Brain access can occur by different mechanisms, including axonal transport and direct or indirect passage through brain barriers (4, 5). Some viruses such as the human immunodeficiency virus (HIV) or West Nile virus (WNV) use the “Trojan horse” mechanism to cross the BBB: this occurs when infected cells of the immune system, with their natural ability to transcytose and reach the brain, allow viruses to penetrate the parenchyma (6, 7). Some viruses can also directly infect BECs or take advantage of the effect of inflammatory cytokines produced systematically upon infection that will transiently impair BBB impermeability and therefore facilitate viral entry to the CNS (4). Similarly, inflammatory chemokines may recruit immune cells, which, if infected, allow more virus to enter, as was proposed for HIV (8). Among neurotropic viruses, several arboviruses (arthropod-borne viruses, mostly responsible for acute infections leading to flu-like symptoms in the majority of symptomatic cases), can also access the CNS and cause a range of pathologies (9). For example, WNV, Japanese encephalitis virus (JEV), Usutu virus, and chikungunya virus are well described as determinants of neurological impairments in some patients, which can be in some cases fatal for the host (9). Recently, Zika virus (ZIKV) reemerged in the Pacific Islands and in South America, causing a major epidemic (10). ZIKV belongs to the *Flaviviridae* family and is a small single-stranded RNA enveloped virus isolated in 1947 in the Zika forest in Uganda (11). Two main lineages exist, namely, the original African and the more recent Asian ones. Interestingly, differences in virulence *in vitro* and in animal models have been reported for the two lineages, suggesting potential clinical difference in humans as well (12). ZIKV is transmitted mainly by the mosquito Aedes aegypti, but other less classical modes for an arbovirus have been described, including blood transfusion, sexual, and mother-to-child transmissions (13). Because of the extent of the American epidemic (more than one million patients were affected), severe forms of the disease were reported, including serious neurological complications such as Guillain-Barré syndrome (GBS) and congenital Zika syndrome (CZS) consisting of microcephaly and other neurodevelopmental defects (10, 13, 14). Regarding CZS, studies demonstrated that ZIKV can cross the blood-placental barrier and replicate in the placenta and in the developing embryo, including the brain, resulting in severe malformations at birth (15). In adults, several neurological symptoms have been described, including encephalitis and meningoencephalitis (16), suggesting that the virus can reach the mature CNS by crossing the BBB.

Several studies are starting to describe the ZIKV neurovirulence molecular and cellular mechanisms (10). One of the key issues remaining, however, is the characterization of the mechanisms governing ZIKV CNS access. Some flaviviruses such as WNV and JEV are known to interact with the BBB, although they use different mechanisms such as direct infection of the endothelial cells or the Trojan horse mechanism allowing transcytosis of infected immune cells (17). Interestingly, inflammatory mediators produced during infection will also have a direct effect on the BBB, either restraining (18) or facilitating CNS access (19). Regarding ZIKV, it was reported using primary human BECs or induced pluripotent stem cell (IPSC)-derived BBB models that Asian ZIKV strains and the original, albeit controversial, MR766 African strain are able to directly infect BECs and replicate without disrupting the BBB integrity *in vitro* (20–23). Studies *in vivo* are inconsistent, since depending on strains and methods used, authors report no effect on the BBB integrity (21) or subtle effects that are strain dependent (23). Studies of BBB infection *in vitro* are particularly challenging, as many BBB models do not fully describe the proper characteristics of the BBB found *in vivo* (24): immortalized cell lines are evidently far from displaying physiologically BBB phenotypes, in particular, because they fail to demonstrate low permeability to small molecules. Primary human BECs can lose their characteristics upon cell passages and display low transendothelial electrical resistance (24). Here, we used a multicellular *in vitro* human BBB model, based on the use of hematopoietic stem cells, which differentiate into brain-like endothelial cells (hBLECs), which display the main characteristics of the human BBB and are stable over an extended period of time, allowing study of long-term effects (25–28). We monitored the infection and effects of an African and an Asian strain of ZIKV on hBLECs and on pericytes, key components of the NVU that are emerging as potent mediators in neuroinflammation (29), as well as on astrocytes. We show that both ZIKV strains can readily infect and replicate in a polarized BBB, with partial effects on its integrity. Nonetheless, chemokines and inflammatory cytokines were produced and secreted both apically and basolaterally upon infection, together with an upregulation of adhesion molecules, which favors immune cell recruitment and docking to the BBB and may play a role in the neuroinflammatory mechanisms associated with this virus. Finally, similar observations were obtained in a mouse model of ZIKV infection as well in plasma samples from ZIKV-infected patients.

## RESULTS

### ZIKV replicates in hBLECs of an *in vitro* human BBB model and in brain pericytes with partial perturbation of endothelial permeability.

**FIG 1:**
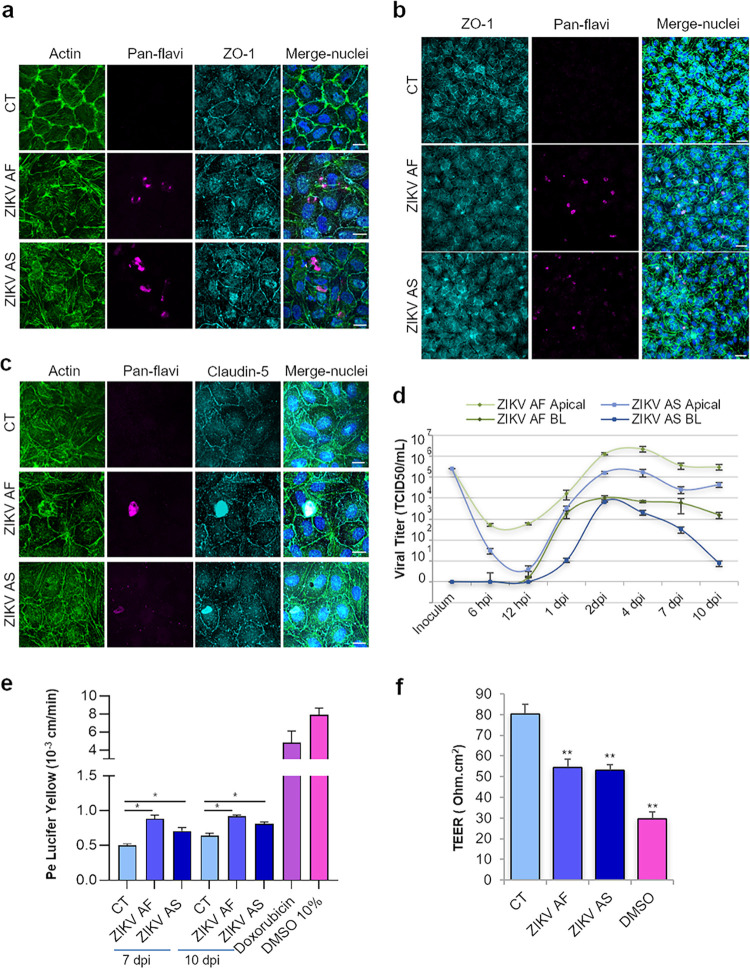
*In vitro* human BBB is permissive to ZIKV infection and replication without strong deleterious effects on its integrity. (a) CT-, ZIKV AF-, and ZIKV AS-infected (MOI, 0.1) hBLECs of BBB model grown on cell culture inserts fixed at 6 dpi. Indirect IF confocal studies of CT- and ZIKV-infected hBLECs using an actin probe (green) and antibodies against ZIKV (pan-flavivirus, magenta) and ZO-1 (cyan). Nuclei are labeled with Hoechst (blue). Bars, 15 μm. (b) CT-, ZIKV AF-, and ZIKV AS-infected (MOI, 1) BBB model grown on cell culture inserts were fixed at 10 dpi. Indirect IF confocal studies show actin (green), ZIKV (magenta), ZO-1 (cyan), and nuclei (blue). ZO-1 labeling highlights cell-cell adhesion, characteristic of polarized endothelia. Bars, 30 μm. (c) Indirect IF studies of BBB model at 7 dpi (MOI, 1) showing actin (green), ZIKV (magenta), claudin-5 (cyan), and nuclei (blue). Bars, 15 μm. (d) Viral titers in supernatants from ZIKV AF- and ZIKV AS-infected (MOI, 1) BBB model in apical and basolateral (BL) sides at various time points postinfection determined using the TCID_50_ method. Results are expressed as means ± standard errors of the means (SEMs) from 3 independent experiments. (e) Paracellular permeability of CT-, ZIKV AF-, and ZIKV AS-infected (MOI, 1) BBB model grown on cell culture inserts at 7 and 10 dpi. Doxorubicin and DMSO are two positive controls. Results are expressed as means ± SEMs (*n* = 3) and analyzed using a Wilcoxon-Mann-Whitney test. *, *P* < 0.05 (ZIKV AF/AS compared to CT). (f) Transendothelial electrical resistance (TEER) of CT-, ZIKV AF-, and ZIKV AS-infected (MOI, 1) BBB grown on cell culture inserts was measured at 10 dpi. DMSO is a positive control. Each bar represents the mean ± SEM from 3 independent experiments and analyzed using a Wilcoxon-Mann-Whitney test. **, *P* < 0.01 (compared to CT).

Viruses can cross the BBB using several pathways, including direct infection of brain microvascular endothelial cells, transcytosis, or the Trojan horse mechanism. To determine whether ZIKV can directly infect the BBB, we incubated a human *in vitro* BBB model that recapitulates the main characteristics of the barrier (25, 30, 31) with different multiplicities of infection (MOIs) of African (ZIKV AF) and Asian (ZIKV AS) strains of ZIKV (MOI, 0.1 and 1). Briefly, CD34^+^ cord blood-derived hematopoietic stem cells were differentiated into endothelial cells and were subsequently seeded on culture inserts with brain bovine pericytes for 5 to 6 days to acquire BBB characteristics and become hBLECs before infections were performed (see Fig. S1a and b in the supplemental material). After initial infection, supernatants were collected and cells fixed at different days postinfection (dpi). Indirect immunofluorescence (IF) studies were then performed using a probe to label actin and an antibody against the junctional-associated protein zonula occludens (ZO)-1 to visualize endothelium architecture and an antibody (pan-flavivirus) to label viruses (Fig. 1a). While viral antigens were detected in cells at 6 dpi (MOI, 0.1), suggesting viral replication (Fig. 1a), global endothelium architecture did not appear massively perturbed, even at 10 dpi and a higher MOI (MOI, 1) (Fig. 1b and c), in contrast to what we previously showed in retinal pigment epithelium, which was very sensitive to ZIKV infection (32). Indeed, the localization of tight junction (TJ) proteins ZO-1 and claudin-5 did not appear strongly affected (Fig. 1b and c). However, the actin cytoskeleton showed rearrangement upon infection, and ZIKV-infected cells appeared to be higher, albeit not detached, in the endothelium as confocal imaging and three-dimensional (3D) reconstruction using imaging software showed, suggesting that some changes in the endothelium morphology may occur (Fig. S1c and d).

10.1128/mBio.01183-20.1 TEXT S1 Supplemental methods. Download Text S1, DOCX file, 0.1 MB. Copyright © 2020 Clé et al. 2020 Clé et al. This content is distributed under the terms of the Creative Commons Attribution 4.0 International license.

10.1128/mBio.01183-20.2 FIG S1 Human BBB is readily infected by ZIKV. (a) Depiction of human blood-brain barrier grown on transwell filters. (b) Table showing the main characteristics of the BBB. (c) CT and ZIKV AF- and ZIKV AS-infected (MOI, 1) hBLECs of BBB model grown on cell culture inserts fixed at 7 dpi. Indirect IF confocal studies of CT and ZIKV-infected hBLECs using an actin probe (green) and antibodies against pan-flavivirus (magenta). Nuclei are labeled with Hoechst (blue). Bars, 10 μm. (d) 3D rendering of mock- and ZIKV-infected BBB. Confocal stacks of images from mock- or ZIKV-infected hBLECs at 7 dpi were subjected to 3D reconstruction with the Imaris software. Actin is colored in green, ZIKV in magenta, and nuclei in blue. Download FIG S1, TIF file, 0.8 MB. Copyright © 2020 Clé et al. 2020 Clé et al. This content is distributed under the terms of the Creative Commons Attribution 4.0 International license.

To have a quantitative approach to monitor viral replication, we measured viral titers in supernatants from ZIKV-infected BBB apical and basolateral sides at various time points using the 50% tissue culture infective dose (TCID_50_) method (Fig. 1d and Fig. S2a). ZIKV AF and ZIKV AS were both efficiently replicating at MOIs of 0.1 and 1 and released from the apical side, which would correspond to the blood vessel luminal side, and from the basolateral side, which would correspond to the parenchymal side (Fig. 1d, MOI, 1; and Fig. S2a, MOI, 0.1). Viral production appeared to decrease slightly over time, but viral replication still occurred at 10 dpi (Fig. 1d). Viral RNA was detected by IF using a specific antibody against double-stranded (ds)-RNA and by a quantitative reverse transcription-PCR (RT-qPCR) approach (Fig. S2b and c), confirming active viral replication until at least 10 dpi. We then characterized whether the endothelium integrity and impermeability were perturbed upon ZIKV hBLEC infection. We used a fluorescence-based assay with Lucifer yellow (LY) to measure the endothelial permeability coefficient, and we measured the transendothelial electrical resistance (TEER) (25). The permeabilities of mock- and ZIKV-infected endothelia at MOIs of 0.1 and 1 were measured at 7 and 10 dpi. Mock-infected (control [CT]) endothelium displayed a permeability coefficient (Pe) of ∼0.5 × 10^−3 ^cm/min, consistent with “tight” BBB endothelia (Fig. 1e). Interestingly, albeit important and efficient viral replication was occurring in ZIKV-infected cells, Pe was increased but still consistent with a tight BBB, suggesting that infection by the two strains of ZIKV did not massively impair BBB integrity but may trigger a subtle effect on the barrier integrity (Fig. 1e). Treatment of cells with doxorubicin, known to destabilize the endothelium, gave a Pe of 4.8 × 10^−3 ^cm/min, while 10% dimethyl sulfoxide (DMSO) treatment led to a Pe of 7.83 × 10^−3 ^cm/min (Fig. 1e). TEER measurement at 10 dpi showed also a decrease in endothelium impermeability in ZIKV-infected BBB, consistent with the LY data (Fig. 1f). However, BBB permeability was not perturbed at a lower MOI (0.1) (Fig. S2d and e). Notably, basolaterally released ZIKV was much lower (ZIKV AF) or absent (ZIKV AS) at an MOI of 0.1 (Fig. S2a) and could potentially (and partially) explain why permeability was not perturbed.

10.1128/mBio.01183-20.3 FIG S2 ZIKV efficiently replicates in hBLECs and pericytes without impairing its integrity. (a) Viral titers in supernatants from ZIKV AF- and ZIKV AS-infected (MOI, 0.1) hBLECs from the BBB models in apical and basolateral sides at 4 and 7 days determined using the TCID_50_ method. Results are expressed as means ± SEMs of 3 independent experiments. (b) CT and ZIKV AF- and ZIKV AS-infected (MOI, 0.1) hBLECs of BBB model grown on cell culture inserts fixed at 7 dpi. Indirect IF confocal studies of CT and ZIKV-infected hBLECs using an actin probe (green) and antibodies against double-strand RNA (magenta) and ZIKV Env (cyan). Nuclei are labeled with Hoechst (blue). Bars, 30 μm. (c) RT-qPCR analyses of ZIKV genome in hBLECs and pericytes from transwell coculture at 7 dpi (MOI 1). (d and e) Paracellular permeability of CT and ZIKV AF- and ZIKV AS-infected (MOI, 0.1) BBB models grown on cell culture inserts at 7 and 10 dpi. Results are expressed as means ± SEMs (*n* = 3) and analyzed using a Wilcoxon-Mann-Whitney test. Download FIG S2, TIF file, 0.9 MB. Copyright © 2020 Clé et al. 2020 Clé et al. This content is distributed under the terms of the Creative Commons Attribution 4.0 International license.

**FIG 2:**
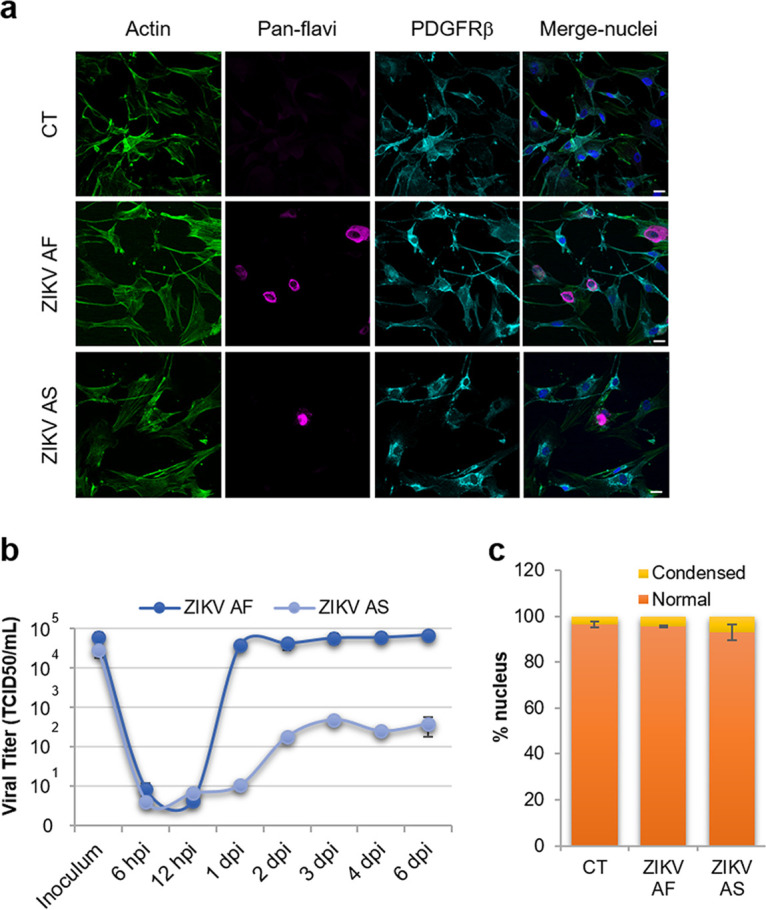
Human pericytes are cellular targets for ZIKV infection. (a) Mock- (CT), ZIKV AF-, and ZIKV AS-infected (MOI, 1) human pericytes were fixed at 4 dpi and labeled with an actin probe (green), pan-flavivirus (magenta), and PDGFRβ (cyan) by indirect IF. Nuclei are labeled with Hoechst (in blue). Bars, 20 μm. (b) Viral titers from ZIKV AF- and ZIKV AS-infected pericytes determined by TCID_50_ methods at various time points postinfection. Results are expressed as means ± SEMs (*n* = 3) and analyzed using a Wilcoxon-Mann-Whitney test. (c) Quantification of apoptotic nuclei in CT-, ZIKV AF-, and ZIKV AS-infected pericytes at 4 dpi. Apoptotic nuclei are represented in yellow and normal nuclei in orange. Results are expressed as means ± SEMs (≥110 cells, *n* = 3).

Because this BBB model consists of coculture of hBLECs and brain bovine pericytes in the basolateral compartment, we also tested for replicating viruses in pericytes using RT-qPCR and specific primers for ZIKV. We therefore collected mRNA from pericytes in coculture with mock and infected hBLECs at 7 dpi and performed RT-qPCR. Interestingly, we detected active replication (Fig. S2c). Because pericytes are starting to gain attention as potential immunomodulators, we then asked whether human pericytes were potential targets during ZIKV CNS infection. Primary human pericytes were infected with ZIKV AF and ZIKV AS at an MOI of 1, and supernatants were harvested at different days postinfection to monitor infectious particle release and fixed at 4 dpi for IF studies. Figure 2a shows ZIKV replication in pericytes as anti-pan-flavivirus antibody labeled some platelet-derived growth factor receptor-positive (PDGFR^+^) cells. Moreover, viral replication quantification with the TCID_50_ method showed that both strains replicated, albeit with different efficiencies (Fig. 2b). It is worthy to note that ZIKV AS poorly replicated, as the titer never exceed 10^3^ TCID_50_/ml at the various days postinfection tested (Fig. 2b). However, ZIKV AF displayed efficient replication with a titer around 10^5^ TCID_50_/ml (Fig. 2b). This viral replication was not associated with cell death/toxicity, as quantification of apoptotic nuclei did not show significant differences in ZIKV-infected pericytes compared to that of mock-infected cells (Fig. 2c). This suggests that the lower replication rate of ZIKV AS in pericytes was not related to cell death.

Together, this set of data suggests that ZIKV directly and efficiently infects the BBB from the apical side and is released from both sides of the endothelium (i.e., can access the parenchyma). Moreover, this release can lead to pericyte infection and to partial perturbation of the BBB integrity.

### ZIKV-infected hBLECs and pericytes upregulate inflammatory cytokines and chemokines.

**FIG 3:**
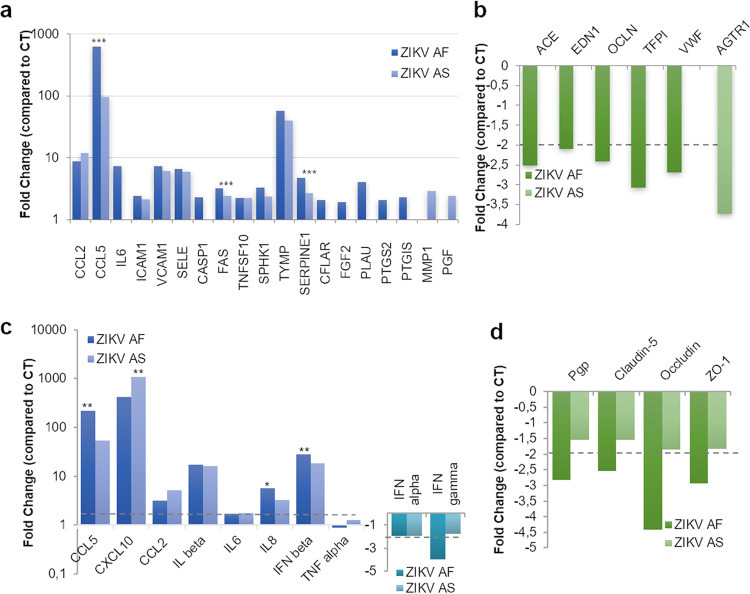
ZIKV infection modulates gene expression in human BBB cells. mRNA from hBLECs from CT-, ZIKV AF-, and ZIKV AS-infected (MOI, 1) BBB model grown on cell culture inserts collected at 7 dpi and subjected to RT-qPCR array analysis. Fold regulation of statistically significant genes upregulated (a) or downregulated (b) in ZIKV AF- and ZIKV AS-infected hBLECs compared to that in CT. Results are expressed as means of the fold change (*n* = 3) (genes where the ratio gene/housekeeping gene is statistically significant from CT) (see Fig. S3c in the supplemental material). Differences between lineages were observed (ratio gene/housekeeping gene ZIKV AF versus ZIKV AS, unpaired *t* test). ***, *P* < 0.001. (c) Gene expression of inflammatory response in hBLECs infected by ZIKV AF and ZIKV AS were measured by RT-qPCR. Results are expressed as means of the fold change (*n* = 3) using *HPRT1* as the housekeeping gene (genes where the ratio gene/housekeeping gene is statistically significant [*P* < 0.05] from CT) (see Fig. S3d). Differences between lineages were observed (ratio gene/housekeeping gene ZIKV AF versus ZIKV AS, unpaired *t* test. *, *P* < 0.05; **, *P* < 0.01. (d) Gene expression of tight junction proteins in hBLECs infected by ZIKV AF and ZIKV AS were measured by RT-qPCR. Results are expressed as means of the fold change (*n* = 3) using *HPRT1* as a housekeeping gene (genes where the ratio gene/housekeeping gene is statistically significant [*P* < 0.05] from CT) (see Fig. S3d).

Because ZIKV readily replicated in our *in vitro* BBB model without strong perturbation of the barrier integrity, we next aimed to monitor whether genes involved in general endothelial homeostasis were modulated upon infection. We first investigated by RT-qPCR the expression of 84 genes involved in endothelial cell biology, including genes regulating cell adhesion, inflammation, injury repair, and angiogenesis (see Materials and Methods). mRNAs from mock-, ZIKV AF-, and ZIKV AS-infected endothelial cells (MOI, 1) were collected at 7 dpi. RT-qPCR analyses then showed the modulation (≥ or ≤2-fold, *P* value ≤ 0.05 compared to CT) (Fig. S3) of several genes in infected cells compared to that under mock-treated conditions (Fig. 3). Twenty-two genes in total were modulated in ZIKV AF-infected cells (17 upregulated, 5 downregulated), whereas ZIKV AS led to the change of expression in 13 genes (12 upregulated, 1 downregulated) (Fig. 3a and b and Fig. S3b). It is interesting to note that although the endothelium integrity was only slightly perturbed, genes of inflammatory mediators such as *CCL2*, *CCL5*, and *IL6* were upregulated by either both strains (*CCL2* and *CCL5*) or only ZIKV AF (*IL6*) (Fig. 3a). Interestingly *VCAM1* and *ICAM1*, encoding two cell adhesion molecules (CAMs) involved in leukocyte docking to the BBB, were also upregulated upon ZIKV infection (Fig. 3a). Other genes encoding proteins involved in adhesion were either upregulated (*SELE*) or downregulated (*VWF*) (Fig. 3a and b). The gene encoding a matrix metalloprotease (*MMP1*) as well as genes involved in the control of apoptosis, such as *FAS*, *CASP1*, or *TNFSF10* (*TRAIL*) were also upregulated, whereas *OCLN*, encoding occludin, a key protein regulating tight junctions, was downregulated by ZIKV AF (Fig. 3a and b). Of note, ZIKV AS led to the downregulation of the angiotensin receptor II gene (*ATGR*), which may play a role in angiogenesis. We then confirmed some of these modulated genes, as well as *CXCL10*, which we found strongly modulated by ZIKV in other cell types (32, 33), by targeted RT-qPCR analyses and found similar modulation (Fig. 3c and d; Fig. S3d). Moreover, we analyzed other genes involved in inflammatory responses (*IL1B*, *IL8*, *TNFA*, *IFNB*, *IFNA*, and *IFNG*) and BBB physiology and found that *IL1B*, *IL8*, and *IFNB* were found upregulated upon infection, whereas *IFNG*, *Pgp*, and genes encoding junctional-associated proteins occludin, claudin 5, and ZO-1 were downregulated in ZIKV AF-infected hBLECs, consistent with the subtle effects on endothelium permeability that we observed (Fig. 3c and d; Fig. S3d).

10.1128/mBio.01183-20.4 FIG S3 Gene modulation in ZIKV-infected hBLECs. (a and b) Volcano plots of genes modulated upon ZIKV infection in hBLECs from the BBB model at 7 dpi normalized to CT expression from Fig. 3a and b. Statistically significant changes in fold regulation appear in the top-right windows (red, genes upregulated) and top-left windows (green; genes downregulated). (c) Statistical analyses from Fig. 3a and b using a Wilcoxon-Mann-Whitney test of comparative cycle threshold (2^−ΔΔ^*^CT^*) (ratio gene/housekeeping gene ZIKV versus CT). (d) Statistical analyses from Fig. 3c and d and Fig. 7a using a Wilcoxon-Mann-Whitney test of 2^−ΔΔ^*^CT^* (ratio gene/housekeeping gene ZIKV versus CT). Download FIG S3, TIF file, 0.5 MB. Copyright © 2020 Clé et al. 2020 Clé et al. This content is distributed under the terms of the Creative Commons Attribution 4.0 International license.

**FIG 4:**
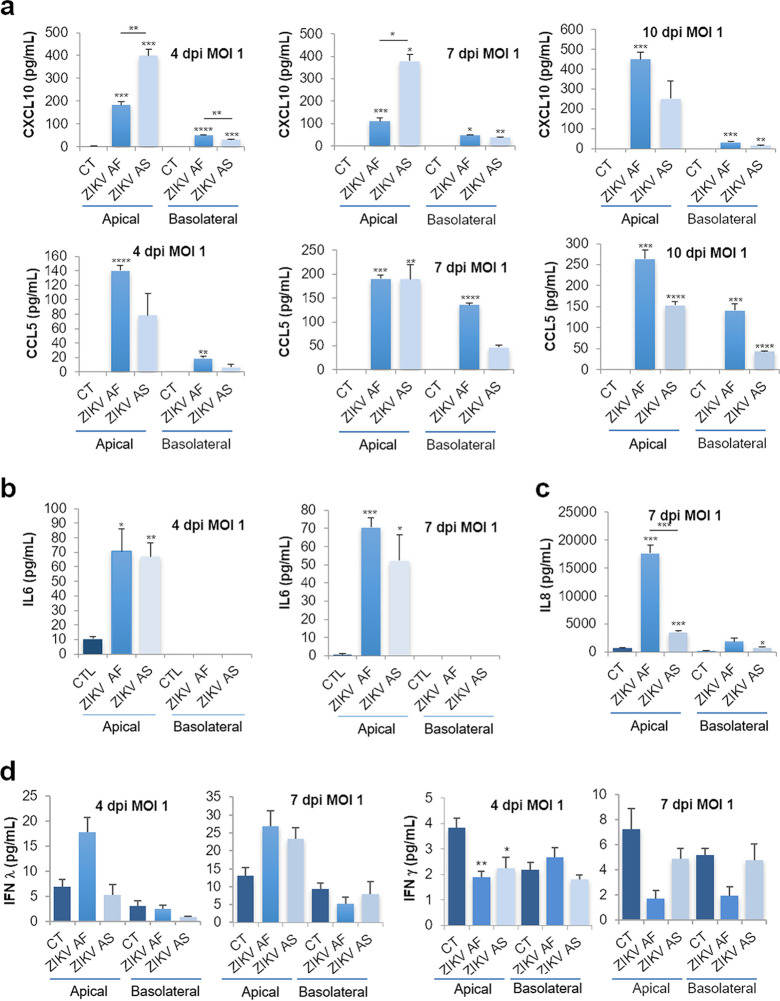
Increased expression of cytokines and chemokines in ZIKV-infected human BBB. (a) ELISA analyses of CXCL10 and CCL5 concentrations in the supernatants (apical and basolateral compartments) of CT or ZIKV AF- and ZIKV AS-infected (MOI, 1) BBB model grown on cell culture inserts at 4, 7, and 10 dpi. Results are expressed as means ± SEMs (*n* = 3) and analyzed using an unpaired *t* test. *, *P* < 0.05; **, *P* < 0.01; ***, *P* < 0.001; ****, *P* < 0.0001 compared to CT. (b) ELISA analyses of IL-6 concentration in the supernatants (apical and basolateral compartments) of CT or ZIKV AF- and ZIKV AS-infected (MOI, 1) BBB model grown on cell culture inserts at 4 and 7 dpi. Results are represented as means ± SEMs (*n* = 3) and analyzed using an unpaired *t* test. *, *P* < 0.05; **, *P* < 0.01; ***, *P* < 0.001 compared to CT. (c) Multiplex analyses of IL-8 concentration in the supernatants (apical and basolateral compartments) of CT or ZIKV AF- and ZIKV AS-infected (MOI, 1) BBB model grown on cell culture inserts at 7 dpi. Results are represented as means ± SEMs (*n* = 3) and analyzed using an unpaired *t* test. *, *P* < 0.05; ***, *P* < 0.001 compared to CT. (d) Multiplex analyses of IFN-γ and -λ concentrations in the supernatants (apical and basolateral compartments) of CT or ZIKV AF- and ZIKV AS-infected (MOI, 1) BBB model grown on cell culture inserts at 4 and 7 dpi. Results are represented as means ± SEMs (*n* = 3) analyzed using an unpaired *t* test. *, *P* < 0.05; **, *P* < 0.01 compared to CT.

Because genes involved in inflammation were modulated, we then monitored the secretion of key cytokines and chemokines, known to modulate antiviral response and immune cell activation/recruitment. Using different approaches, we measured the concentrations in apical and basolateral compartments of CXCL10, interleukin 6 (IL-6), CCL5, CCL2, IL-8, and interferon (IFN)-α, -β, -γ, and -λ. At both MOIs (Fig. 4 and Fig. S4), the expression and secretion of some cytokines and chemokines appeared to be increased in ZIKV-infected hBLECs, in the apical and sometimes basolateral compartments. Figure 4 shows that both strains led to increased expression/secretion of CXCL10, IL-6, and CCL5. Interestingly, as we and others showed that ZIKV AF strains were in most of the case more virulent than Asian strains of ZIKV in various cellular systems and *in vivo* (12), some cytokines and chemokines were found differentially modulated by the two strains, in particular, CXCL10 and IL-8 (Fig. 4a and c). To measure IFN production, we use a multiplex assay aimed to analyze IFN-α, -β, -γ, and -λ concentrations in both compartments. Unfortunately, with this assay, we only detected IFN-γ and -λ and showed that, similarly to what we observed in RT-qPCR analyses, IFN-γ was downregulated (Fig. 4d). IFN-λ however, seemed to be slightly upregulated by both strains, albeit not significantly (Fig. 4d).

10.1128/mBio.01183-20.5 FIG S4 Increased expression of cytokines and chemokines in ZIKV-infected human BBB. ELISA analyses of CXCL10, CCL5, IL-6, and CCL2 concentrations in the supernatants (apical and basolateral compartments) of CT or ZIKV AF- and ZIKV AS-infected (MOI, 0.1) BBB models grown on cell culture inserts at 4 and 7 dpi. Results are expressed as means ± SEMs (*n* = 3) and analyzed using an unpaired *t* test. *, *P* < 0.05; **, *P* < 0.01; ***, *P* < 0.001; ****, *P* < 0.0001 ZIKV AF or AS compared to CT. Download FIG S4, TIF file, 0.1 MB. Copyright © 2020 Clé et al. 2020 Clé et al. This content is distributed under the terms of the Creative Commons Attribution 4.0 International license.

**FIG 5:**
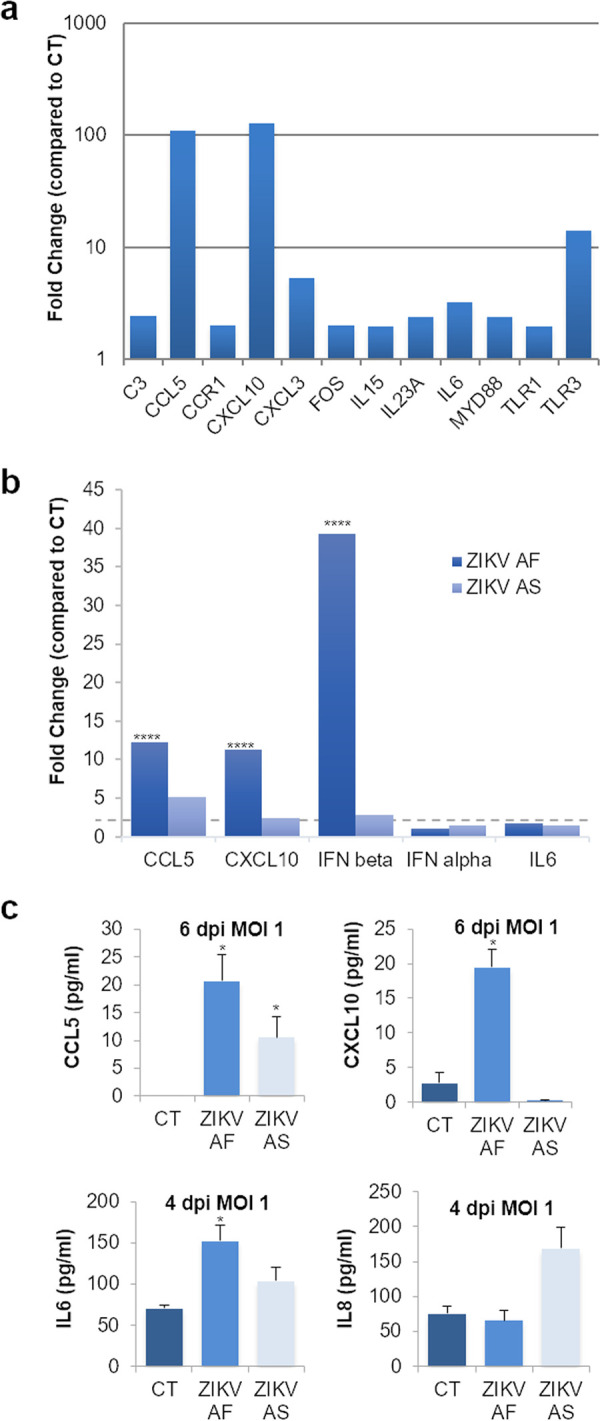
ZIKV-infected pericytes express inflammatory cytokines and chemokines. mRNAs from mock- and ZIKV-infected pericytes (MOI, 1) at 3 dpi were collected and subjected to RT-qPCR analyses using a PCR array of 84 genes implicated in innate and adaptive immunity (see Materials and Methods). (a) Fold regulation of statistically significant genes modulated upon ZIKV AF infection are shown. Only genes where the ratio gene/housekeeping gene were statistically significant (unpaired *t* test *P* < 0.05. ZIKV AF compared to CT) (see Fig. S5a) from CT are shown. Results are expressed as mean ± SEM (*n* = 3). (b) Gene expression of inflammatory response in ZIKV AF and ZIKV AS-infected pericytes (MOI 1) by RT-qPCR at 6 dpi. Results are expressed as mean of the fold change (*n* = 3) using *HPRT1* as housekeeping gene (genes where the ratio gene/housekeeping gene is statistically significant (*P* < 0.05) from CT (see Fig. S5b). Differences between lineages were observed (ratio gene/housekeeping gene ZIKV AF versus ZIKV AS, unpaired *t* test) ****, *P* < 0.0001. (c) ELISA and multiplex analyses of CXCL10, CCL5, IL-6, and IL-8 concentrations in the supernatants of CT or ZIKV AF- and ZIKV AS-infected (MOI, 1) primary human pericytes at 4 and 6 dpi. Results are represented as means ± SEMs (*n* = 3) and analyzed using an unpaired *t* test. *, *P* < 0.05 compared to CT.

We then monitored the modulation of genes involved in inflammation and immunity in ZIKV-infected human pericytes. We performed RT-qPCR analyses on a panel of 84 genes involved in pathways regulating inflammatory responses (see Materials and Methods) or for targeted genes in mock-, ZIKV AF-, and ZIKV AS-infected brain pericytes at an MOI of 1 and collected the mRNA at 3 and 6 dpi. Figure 5A shows gene modulation (≤2-fold, *P* value ≤ 0.05 compared to CT) (Fig. S5a) by ZIKV AF at an early time of infection (3 dpi). Similarly to that in hBLECs, the chemokines CCL5 and CXCL10 were upregulated by ZIKV (Fig. 5a). As described in many other cell types during the course of ZIKV infection, Toll-like receptor 3 (TLR3) gene transcription was also increased in ZIKV-infected pericytes along with genes for the proinflammatory cytokines IL-6 and IL-15 (Fig. 5a). We next analyzed by single-gene assay ZIKV AF- and ZIKV AS-infected pericytes at 6 dpi. Both *CCL5* and *CXCL10* were upregulated by the two strains, as well as *IFNB* (Fig. 5b). *IL6* modulation was just under the 2-fold threshold compared to that for the CT but was significantly upregulated (Fig. 5b; Fig. S5b). Enzyme-linked immunosorbent assays (ELISAs) on mock- and ZIKV-infected supernatants collected at 4 and 6 dpi showed increases in the expression levels of CCL5 (both strains), CXCL10, and IL-6 (ZIKV AF), as well as potentially IL-8 (ZIKV AS, nonsignificantly, however) (Fig. 5c).

**FIG 6:**
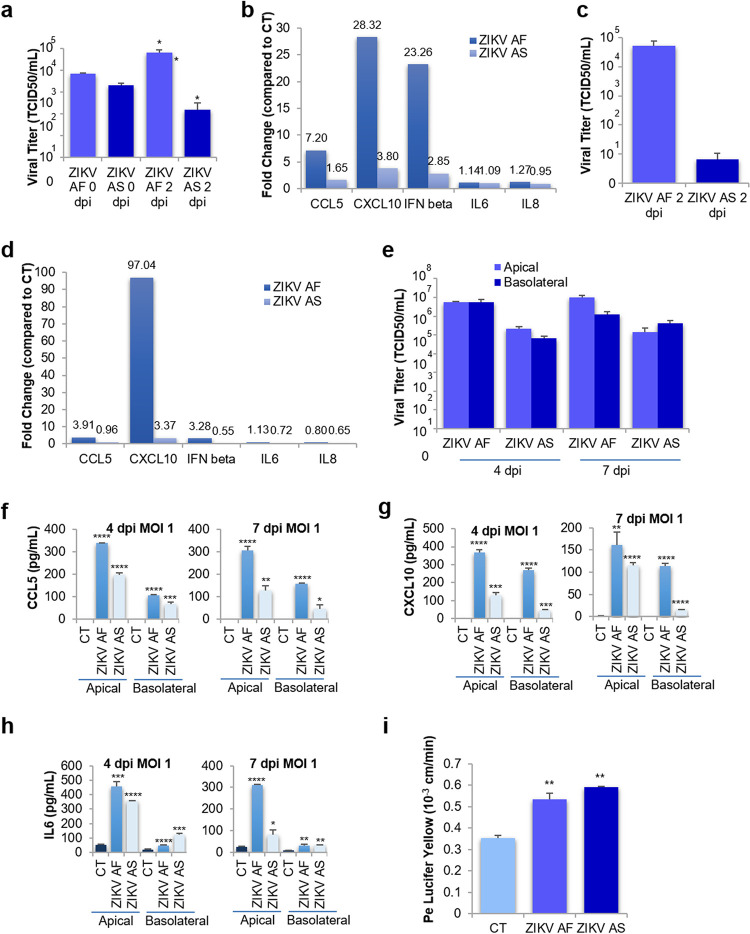
ZIKV triggers inflammatory responses in human astrocytes. (a) Basolateral supernatants from ZIKV AF- and ZIKV AS-infected BBB (hBLECs plus pericytes) at 4 dpi were incubated with astrocytes for 2 days. Viral titers from ZIKV AF- and ZIKV AS-infected astrocytes were determined by TCID_50_ at various times postinfection. Results are expressed as means ± SEMs (*n* = 3) and analyzed using a Wilcoxon-Mann-Whitney test. *, *P* < 0.05 (ZIKV AF versus AS). (b) Gene expression of inflammatory response in astrocytes infected by basolateral supernatants from ZIKV AF- and ZIKV AS-infected BBB (hBLECs plus pericytes) were measured by qRT-PCR. Results are expressed as means of the fold change (*n* = 3) using *HPRT1* as a housekeeping gene (genes where the ratio gene/housekeeping gene is statistically significant [*P* < 0.05] from CT) (see Fig. S5c). Differences between lineages were observed (ratio gene/housekeeping gene ZIKV AF versus ZIKV AS, unpaired *t* test). (c) Viral titers from ZIKV AF- and ZIKV AS-infected astrocytes were determined by TCID_50_ at 2 dpi. Results are expressed as means ± SEMs (*n* = 3) and analyzed using a Wilcoxon-Mann-Whitney test. (d) Gene expression of inflammatory response in ZIKV AF- and ZIKV AS-infected astrocytes was measured by qRT-PCR. Results are expressed as means of the fold change (*n* = 3) using *HPRT1* as a housekeeping gene (genes where the ratio gene/housekeeping gene is statistically significant [*P* < 0.05] from CT) (see Fig. S5d). Differences between lineages were observed (ratio gene/housekeeping gene ZIKV AF versus ZIKV AS, unpaired *t* test. (e) Viral titers in supernatants from ZIKV AF- and ZIKV AS-infected (MOI, 1) BBB model (hBLECs/pericytes/astrocytes) in apical and basolateral sides at 4 and 7 dpi determined using the TCID_50_ method. Results are expressed as means ± SEMs from 3 independent experiments. ELISA analyses of CCL5 (f), CXCL10 (g), and IL-6 (h) concentrations in the supernatants (apical and basolateral compartments) of CT or ZIKV AF- and ZIKV AS-infected (MOI, 1) BBB model grown (hBLECs/pericytes/astrocytes) on cell culture inserts at 4 and 7 dpi. Results are expressed as means ± SEMs (*n* = 3) and analyzed using a nonparametric *t* test. *, *P* < 0.05; **, *P* < 0.01; ***, *P* < 0.001; ****, *P* < 0.0001 compared to CT. (i) Paracellular permeability of CT or ZIKV AF- and ZIKV AS-infected (MOI, 1) BBB model (hBLECs/pericytes/astrocytes) grown on cell culture inserts at 7 dpi. Results are expressed as means ± SEMs (*n* = 3) and analyzed using a Wilcoxon-Mann-Whitney test. **, *P* < 0.01 (ZIKV AF/AS compared to CT).

10.1128/mBio.01183-20.6 FIG S5 Gene modulation in ZIKV-infected pericytes and astrocytes. (a) Statistical analyses from Fig. 5a using a Wilcoxon-Mann-Whitney test of 2^−ΔΔ^*^CT^* (ratio gene/housekeeping gene ZIKV versus CT). (b) Statistical analyses from Fig. 5b using a Wilcoxon-Mann-Whitney test of 2^−ΔΔ^*^CT^* (ratio gene/housekeeping gene ZIKV-AF and ZIKV-AS versus CT). (c) Statistical analyses from Fig. 6b using a Wilcoxon-Mann-Whitney test of 2^−ΔΔ^*^CT^* (ratio gene/housekeeping gene ZIKV-AF, ZIKV-AS versus CT). (d) Statistical analyses from Fig. 6d using a Wilcoxon-Mann-Whitney test of 2^−ΔΔ^*^CT^* (ratio gene/housekeeping gene ZIKV-AF, ZIKV-AS versus CT). (e) Description of human blood-brain barrier grown on transwell filters (endothelial cells in red, pericytes in purple, and astrocytes in blue). Download FIG S5, TIF file, 0.1 MB. Copyright © 2020 Clé et al. 2020 Clé et al. This content is distributed under the terms of the Creative Commons Attribution 4.0 International license.

Altogether, this set of data showed that some of the BBB proteins are modulated by ZIKV infection in hBLECs and pericytes, even though the BBB integrity is not strongly perturbed. Proteins involved in inflammatory responses and chemoattraction as well as tight junction (TJ) proteins and adhesion molecules are affected upon infection and may trigger the recruitment of cells of the immune system and promote local inflammation.

### Astrocytes may potentiate local ZIKV BBB replication and inflammatory response.

As astrocytes are known to be targeted during ZIKV brain infection, as we and others demonstrated (33, 34), and because astrocytes are involved in the maintenance of BBB integrity (2), we then asked whether ZIKV BBB infection could be affected by this cell type. To monitor whether basolaterally released ZIKV and inflammatory molecules that we found to be modulated (Fig. 4) could affect human primary astrocytes, we incubated cells with basolateral supernatants from ZIKV-infected BBB (hBLECs plus pericytes) at 4 dpi for 2 days (final MOIs: ZIKV AF, 0.059; ZIKV AS, 0.007). We first measured whether further replication occurred and showed that ZIKV AF indeed replicated, as the initial titer increased (Fig. 6a). However, ZIKV AS did not lead to important replication in these time windows (Fig. 6a). mRNAs were extracted and RT-qPCRs on selected inflammatory genes were performed. Figure 6b shows that *CCL5*, *CXCL10*, and *IFNB* were modulated by both strains, albeit with significant differences between ZIKV AF and ZIKV AS (Fig. 6b and Fig. S5c). Because this genetic modulation of inflammatory molecules could be the result of ZIKV astrocyte infection and/or the effect of already present cytokines and chemokines in the basolateral compartment, we then directly infected human astrocytes with the same MOI in the basolateral compartments and measured viral titer and increase in inflammatory molecules at 2 dpi. Viral titers showed efficient replication of ZIKV AF, in a similar range as observed with incubation of the basolateral compartment, but poor replication of ZIKV AS (Fig. 6c). Regarding the modulation of inflammatory cytokines, only *CXCL10* was found to be more modulated by direct infection than by incubation with basolateral supernatants (Fig. 6d and Fig. S5d), suggesting that the combination of viral particles and cytokines and chemokines released by the basolateral side of hBLECs can potentiate the inflammatory responses in astrocytes.

Finally, we performed ZIKV infection of a triple-culture BBB model, where pericytes are cultured at the bottom of the transwell filter, coculture with hBLECs is allowed for 7 days prior to infection (MOI, 1), and human primary astrocytes are added to the wells (35) (Fig. S5e). Four and 7 dpi, we measured viral replication in apical and basolateral supernatants. Apical viral titers were in the same range as was observed in the coculture model (Fig. 6e and Fig. 1d), whereas basolateral titers were significantly higher (1 to 2 log), possibly due to active replication in astrocytes (Fig. 6e and Fig. 1d). ELISA analyses performed in apical and basolateral compartments showed increased expression of CXCL10, CCL5, and IL-6 (Fig. 6f to h). Notably, basolateral levels of these cytokines were higher and found earlier than in the coculture system (Fig. 4). However, BBB permeability measurements showed similar modulation by ZIKV infection as was found in coculture (Fig. 6i and Fig. 1e), suggesting that ZIKV infection/modulation of astrocytes, albeit increasing the inflammatory environment, did not further perturb BBB integrity in this model.

Together, these data suggest than ZIKV released from hBLECs leads to efficient infection of astrocytes and potentiates the inflammatory environment.

### ZIKV modulates CAM expression and favors leukocyte recruitment to the BBB.

**FIG 7:**
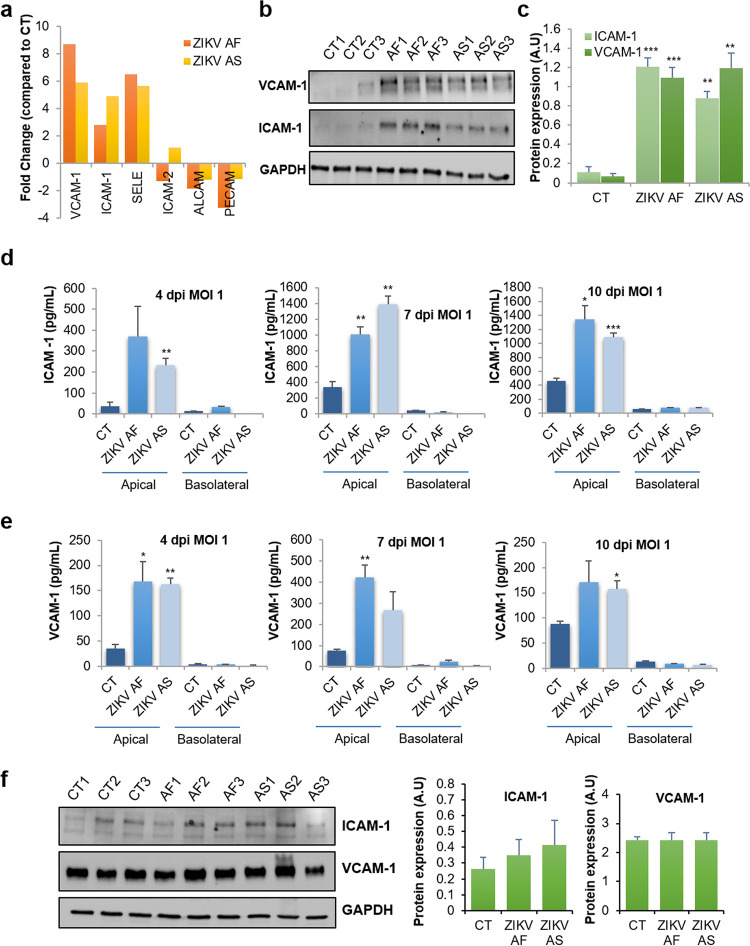
ZIKV hBLEC infection triggers strong upregulation of cell adhesion molecules. (a) Gene expression of cell adhesion molecules (ICAM-1, VCAM-1, E-selectin [SELE], ICAM-2, ALCAM-2, and PECAM) in hBLECs infected by ZIKV AF and ZIKV AS. mRNA from hBLECs from CT or ZIKV AF- and ZIKV AS-infected (MOI, 1) BBB model grown on cell culture inserts were collected at 7 dpi and subjected to RT-qPCR array analyses. Results are expressed as means of the fold change (*n* = 3) using *HPRT1* as a housekeeping gene (genes where the ratio gene/housekeeping gene is statistically significant [*P* < 0.05] from CT) (see Fig. S3d). (b and c) Immunoblot blot analyses of the expression of ICAM-1 and VCAM-1 in CT or ZIKV AF- and ZIKV AS-infected hBLECs at 7 dpi (MOI, 1). Representative images are shown. The quantification of the expression of these markers, relative to GAPDH expression, is expressed as mean ± SEM (*n* = 3) and analyzed using a Student's *t* test. **, *P* < 0.01; ***, *P* < 0.001 compared to CT. (d and e) ELISA analyses of soluble ICAM-1 and VCAM-1 concentrations in the supernatants (apical and basolateral compartments) of CT or ZIKV AF- and ZIKV AS-infected (MOI, 1) hBLECs of the BBB model grown on cell culture inserts at 4, 7, and 10 dpi. Results are expressed as means ± SEMs (*n* = 3) and analyzed using a Student's *t* test. *, *P* < 0.05; **, *P* < 0.01; ***, *P* < 0.001 compared to CT. (f) Immunoblot analysis of the expression of CAMs in mock-, ZIKV AF-, and ZIKV AS-infected primary human pericytes at 6 dpi with an MOI of 1. Representative images are shown. The quantification of the expression of these markers, relative to GAPDH expression, is expressed as means ± SEMs from 3 experiments.

Because we detected the upregulation of some CAMs in our RT-qPCR array that are classically involved in leukocyte binding to the BBB endothelial cells, such as ICAM-1, SELE, and VCAM-1 (36), we then aimed to confirm these observations in single-gene assays. RT-qPCR analyses using single sets of primers showed the genetic modulation of *VCAM1*, *ICAM1*, *SELE*, and genes encoding other CAMs known to be expressed on the BBB. We observed that ZIKV infection led to selective upregulation of CAMs (*VCAM1*, *ICAM1*, and *SELE*) whereas *PECAM* was downregulated upon ZIKV AF infection (Fig. 7a) (≤2-fold, *P* value ≤ 0.05 compared to CT) (Fig. S3d), confirming the data obtained previously with the array (Fig. 3a). We then monitored VCAM-1 and ICAM-1 expression by immunoblotting under similar conditions and found strong upregulation by the two strains (Fig. 7b and c). Because the extracellular domains of CAMs can be released from cells and act as soluble factors, we measured soluble CAM (sCAM) concentrations in the apical and basolateral compartments of mock- and ZIKV-infected hBLECs at various days postinfection. Figure 7d and e show that both soluble ICAM-1 and VCAM-1 are released by infected hBLECs from 4 to 10 dpi. Interestingly, the modulation of CAMs seemed to be specific for hBLECs, as ZIKV-infected pericytes did not show an increase of total or secreted levels of these proteins (Fig. 7f).

**FIG 8:**
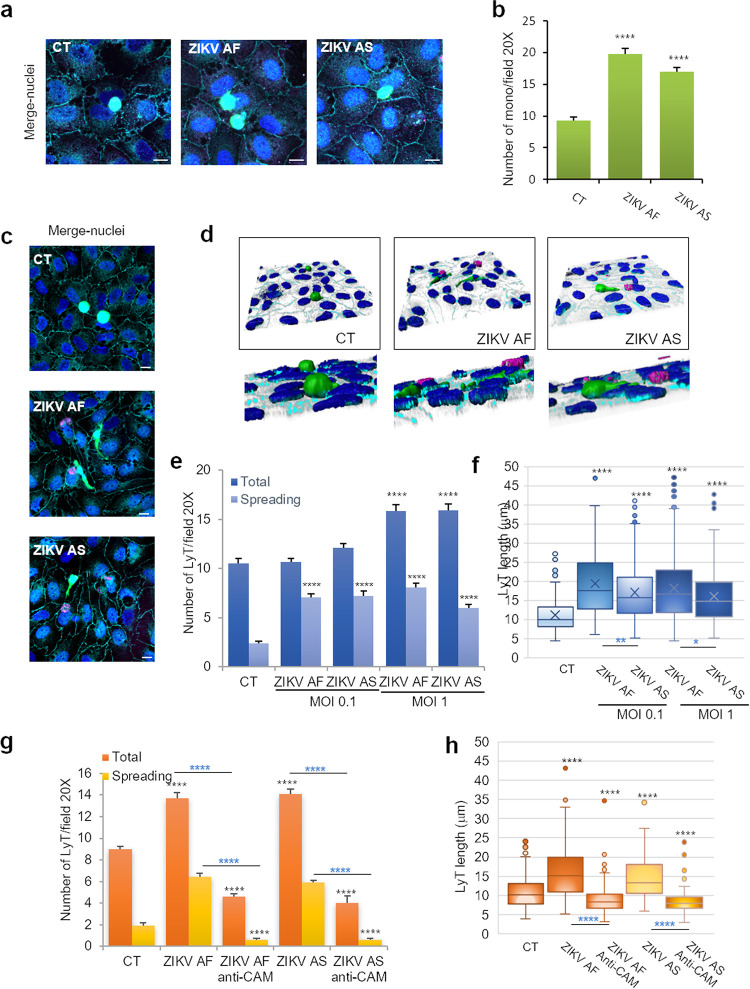
Increased recruitment of leukocytes in ZIKV-infected human BBB. hBLECs grown on cell culture inserts were infected with ZIKV AF and ZIKV AS (MOI, 1 or 0.1) for 7 days; 10^4^ monocytes (a) or lymphocytes CD4^+^ (LyT) (c) prestained with CFSE were added to hBLECs for 30 min. (a) Mock-, ZIKV AF-, and ZIKV AS-infected BBB models grown on cell culture insert were fixed after incubation with monocytes at 7 dpi. Indirect IF studies were used to visualize monocyte (green) interaction with hBLECs: merged image shows also ZIKV (pan-flavivirus, magenta), ZO-1 (cyan) and Hoechst (blue). Scale bars 10 μm. (b) Quantitative analyzes of monocyte numbers per field (20×). Results are expressed mean ± SEM (30 fields per conditions per experiments (≥450 cells, *n* = 3)) and analyzed using a Wilcoxon-Mann-Whitney test. ****, *P* < 0.0001 compared to CT. (c) Mock- and ZIKV AF- and ZIKV AS-infected BBB model grown on cell culture insert were fixed after incubation with LyT at 7 dpi. Indirect IF studies were used to visualize LyT (green) interaction with hBLEC: merge images show also ZIKV (pan-flavivirus, magenta), ZO-1 (cyan), and Hoechst (blue). Bars, 10 μm. (d) 3D rendering of LyT interaction with mock-, ZIKV AF-, and ZIKV AS-infected BBB models. Confocal stacks of images shown in panel c were subjected to 3D reconstruction with the Imaris software (ZO-1, cyan; LyT, green; ZIKV, magenta; and nuclei, blue). (e) Quantitative analyses of LyT numbers per field (20×) in CT and ZIKV-infected BBB (MOI, 0.1 and 1). Cells elongated qualified as “spreading.” Results are expressed means ± SEMs (30 fields per conditions per experiments [≥320 cells, *n* = 3]) and analyzed using a Wilcoxon-Mann-Whitney test. ****, *P* < 0.0001 compared to CT. (f) LyT cell length (in microns) in CT and ZIKV-infected BBB (MOI, 0.1 and 1). Data are expressed as boxes and whiskers (≥300 cells, *n* = 3) and analyzed using a Wilcoxon-Mann-Whitney test. *, *P* < 0.05; **, *P* < 0.01; ****, *P* < 0.0001. Black asterisks show differences compared to CT conditions, while blue asterisks show differences between ZIKV AF and ZIKV AS. (g) To monitor CAM involvement in LyT binding/recruitment, hBLECs were incubated with a cocktail of blocking antibodies against ICAM-1, VCAM-1, and E-selectin 1 h prior to LyT incubation. Quantitative analyses of LyT numbers per field (20×) in CT and ZIKV-infected BBB (MOI, 1). Results are expressed means ± SEMs (30 fields per conditions per experiments [*n* = 3]) and analyzed using a Wilcoxon-Mann-Whitney test. ****, *P* < 0.0001 compared to CT. Black asterisks show differences compared to CT conditions, while blue asterisks show differences between ZIKV AF/AS and ZIKV AF/AS treated with anti-CAM antibodies. (h) LyT cell length (in microns) in CT and ZIKV-infected BBB (MOI, 1) after CAM blocking. Results are expressed means ± SEMs (30 fields per conditions per experiments [*n* = 3]) and analyzed using a Wilcoxon-Mann-Whitney test. ****, *P* < 0.0001 compared to CT. Black asterisks show differences compared to CT conditions, while blue asterisks show differences between ZIKV AF/AS and ZIKV AF/AS treated with anti-CAM antibodies.

We next monitored whether leukocyte recruitment (binding) was affected in ZIKV-infected hBLECs. According to previously published studies (26, 28, 31), we incubated ZIKV-infected BBB at 7 dpi with 10^4^ monocytes or CD4^+^ T cells (LyT) for 30 min and gently washed and fixed them for IF studies. Prior labeling of leukocytes with carboxyfluorescein succinimidyl ester (CFSE) allowed us to visualize monocyte and LyT binding at low (20×) (see Fig. S6a and b) or high (63×) (Fig. 8a and c) magnification. No gross effect on monocyte morphology was observed upon binding to the ZIKV-infected BBB (Fig. 8a). However, quantification of random fields at ×20 magnification showed that ZIKV infection led to significantly more monocyte recruitment (Fig. 8b). When bound LyT were observed, however, striking cellular morphological changes were detected by IF studies and software 3D rendering. Whereas LyT bound to the CT BBB were mostly round, LyT attached to ZIKV-infected hBLECs appeared flatter and to spread more (Fig. 8c and d). Quantification of this “spread” phenotype showed a significant increase under ZIKV infection conditions (Fig. 7e). To have a more quantifiable parameter, cell diameter/length was measured: LyT in contact with ZIKV-infected hBLECs showed increased length (Fig. 8f). We then monitored CAM localization during leukocyte binding: we detected by IF strong labeling of ICAM-1 in the ZIKV-infected BBB, especially in ZIKV-infected cells and in close proximity of monocytes, confirming both RT-qPCR and immunoblot studies (Fig. S6c). Similarly for monocytes, strong ICAM-1 labeling was detected in close proximity to LyT, possibly reflecting CAM recruitment at contact sites (Fig. S6d). To correlate more conclusively CAM upregulation by ZIKV infection to leukocyte recruitment, we then performed a blocking experiment using a cocktail of anti-ICAM-1, -VCAM-1, and E-selectin blocking antibodies as previously described (37). Antibodies were added 1 h prior to incubation with LyT in CT- and ZIKV-infected (MOI, 1) BBB models at 7 dpi. After LyT incubation for 30 min, filters were fixed and processed for IF and confocal analyses. Figure 8g and h show that in the presence of blocking antibodies, both the number of cells (Fig. 8g) and the cell diameter (Fig. 8h) were efficiently decreased, confirming the involvement of these CAMs in LyT recruitment at the BBB.

10.1128/mBio.01183-20.7 FIG S6 Leukocyte binding to mock- and ZIKV-infected in hBLECs. Monocytes (a) or lymphocytes (b) T CD4^+^ (LyT) labeled with CFSE (in green) binding to CT or ZIKV AF- and ZIKV AS-infected (MOI, 0.1) BBB models grown on cell culture inserts 7 dpi. Nuclei are false colored in magenta after Hoechst staining. Bars, 50 μm. (c and d) ZIKV AF- and AS-infected BBB models grown on cell culture inserts were fixed at 7 dpi. Indirect IF studies were used to label monocytes (c) and LyT (d) (green), ZIKV (pan-flavivirus, magenta), ICAM-1 (cyan), and nuclei (Hoechst, blue). Bars, 10 μm (c) and 20 μm (d). Download FIG S6, TIF file, 1.0 MB. Copyright © 2020 Clé et al. 2020 Clé et al. This content is distributed under the terms of the Creative Commons Attribution 4.0 International license.

Altogether, these data suggest that upregulation of CAMs triggered by ZIKV infection may favor leukocyte recruitment to the BBB.

### CAM levels are increased during ZIKV infection in a mouse model and in humans.

**FIG 9:**
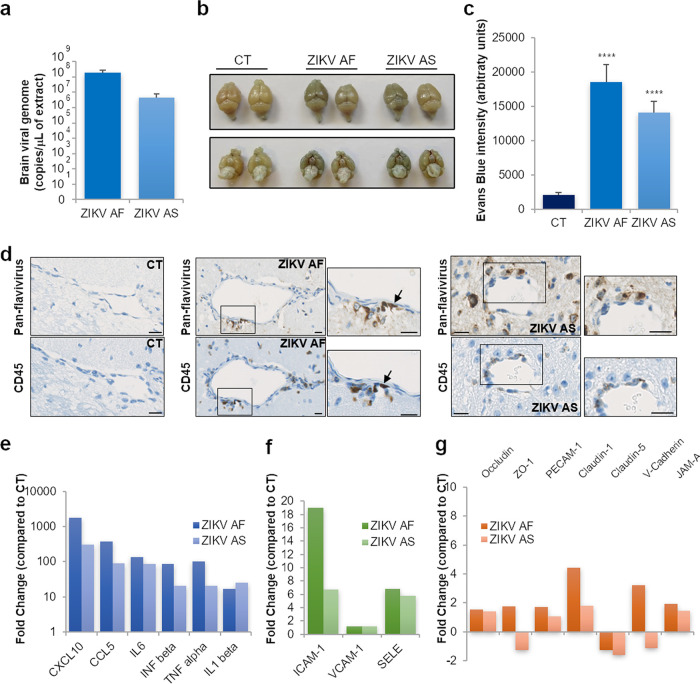
ZIKV-infected mouse brain displays local BBB impairment, leukocyte infiltration, and CAM upregulation. (a) RT-qPCR analyses of ZIKV genome in the brain of ZIKV-infected *Ifnar*^−/−^ mice 7 dpi. (b) Picture of dissected brains from EB-injected mock- and ZIKV-infected mice at 7 dpi. (c) Quantification Evans blue fluorescence in brain slices from mock- and ZIKV-infected animals. Results are expressed means ± SEMs (*n* = 3) and analyzed using a Wilcoxon-Mann-Whitney test. ****, *P* < 0.0001 compared to CT. (d) Three-micron consecutive paraffin sections were processed with Luxol blue and stained either with an anti-pan-flavivirus or an anti-CD45 (lymphoid cells) (brown labeling) antibody. Bars. 10 μm. (e) RT-qPCR analyses of inflammatory genes in ZIKV-infected brains. Results are expressed as means of the fold change (*n* = 3) using *GAPDH* as a housekeeping gene (genes where the ratio gene/housekeeping gene is statistically significant [*P* < 0.05] from CT) (see Fig. S8a). (f) RT-qPCR analyses of CAMs in ZIKV-infected brains. Results are expressed as means of the fold change (*n* = 3) using *GAPDH* as a housekeeping gene (genes where the ratio gene/housekeeping gene is statistically significant [*P* < 0.05] from CT) (see Fig. S8a). (g) RT-qPCR analyses of TJ and AJ genes in ZIKV-infected brains. Results are expressed as means of the fold change (*n* = 3) using *GAPDH* as a housekeeping gene (genes where the ratio gene/housekeeping gene is statistically significant [*P* < 0.05] from CT) (see Fig. S8a).

Next, we analyzed CAM expression in a described mouse model of ZIKV infection and existing human cohorts from the French West Indies to investigate whether this modulation of CAMs during ZIKV (brain) infection was found *in vivo* and in patients. First, we took advantage of the pathogen-free *Ifnar^−/−^* mouse model, which is a pertinent model to study ZIKV pathogenesis (e.g., see reference 38) and that we recently described for ZIKV-related retinal pathology (32). To study BBB integrity and leukocyte CNS infiltration, mice were inoculated via the intraperitoneal route (i.p.) with phosphate-buffered saline (PBS; mock), ZIKV AF, or ZIKV AS (10^4^ TCID_50_/ml per mouse) and euthanized at 7 dpi. Some animals were then subjected to Evans blue (EB; a colorant used to monitor BBB integrity) i.p. injection at 7 dpi and euthanized 6 h post-EB i.p. injection. First, we showed efficient ZIKV brain infection through the detection of viral genomes by RT-qPCR using ZIKV NS5-specific primers (Fig. 9a). Figure 9b shows representative brains after EB and mock or ZIKV infection. Albeit we did not detect strong brain EB labeling as observed following WNV infections (19), ZIKV-infected brain appeared darker than CT brain, suggesting that partial BBB impairment occurred under these conditions (Fig. 9b). EB fluorescence was found sparsely in brain slices and was quantified in mock- and ZIKV-infected animals (see Fig. S7a). However, we showed a significant increase of EB signals in ZIKV-infected brains (Fig. 9c and Fig. S7a). Moreover, histoimmunochemistry showed CD45^+^ (lymphoid cells) and CD3^+^ (T cells) staining, highlighting CNS immune infiltration in ZIKV-infected animals (Fig. S7b). Histoimmunochemistry staining with the pan-flavivirus antibody revealed in ZIKV-infected animals the presence of positive cells lining blood vessels, consistent with cerebral endothelial cell infection by ZIKV (Fig. 9d and Fig. S7d). Moreover, staining with CD45 in consecutive slices (3-μm thick) showed the recruitment of leukocytes at the BBB, some of them positive for ZIKV (Fig. 9d, arrows). To analyze the genetic modulation of neuroinflammation markers, TJ and adherens junction (AJ) proteins, as well as the levels of CAMs that we identified modulated by ZIKV *in vitro*, we performed RT-qPCR assays on several genes. Figure 9e shows that ZIKV-infected brains display an upregulation in the inflammatory genes *CXCL10*, *CCL5*, *TNFA, INFB*, *IL1B*, and *IL6*. Moreover, we observed that *ICAM1* and *SELE*, but not *VCAM1*, were also upregulated, partly consistent with our data with the human BBB *in vitro* model (Fig. 9f). However, genetic analyses of several TJ and AJ genes did not show strong modulation in ZIKV-infected brain (Fig. 9g). Only claudin-1 and V-cadherin were upregulated in ZIKV AF-infected brains. Immunohistological analyses of claudin-5 and ZO-1 expression and localization did not show obvious changes in the BBB area (see Fig. S8b and c), suggesting that BBB integrity was not massively perturbed in these animals but possibly in discrete areas, thus correlating with the *in vitro* results obtained with the hBLECs (Fig. 1).

10.1128/mBio.01183-20.8 FIG S7 ZIKV-infected mouse brain displays local BBB impairment. (a) Evans blue fluorescence (red) and nuclei (blue) in brain slices from CT and ZIKV-infected animals. (b and c) Three-micron paraffin sections brain from mock- and ZIKV-infected mice were processed with Luxol blue and stained either with an anti-CD45 (total immune cells) (b) or anti-CD3 (T lymphocytes) (brown labelling) antibody. (d) Three-micron paraffin sections brain from mock- and ZIKV-infected mice were processed with Luxol blue and stained with anti-pan-flavivirus (brown labelling). Bars, 50 μm and 10 μm in zoomed inserts. Download FIG S7, TIF file, 1.8 MB. Copyright © 2020 Clé et al. 2020 Clé et al. This content is distributed under the terms of the Creative Commons Attribution 4.0 International license.

10.1128/mBio.01183-20.9 FIG S8 ZIKV induces modulation of inflammatory molecules, cells adhesion molecules, and junction protein expression in ZIKV-infected mice. (a) Gene expression of inflammatory molecules, junction molecules, and CAMs in *Ifnar^−/−^* mice infected by ZIKV AF and ZIKV AS. Statistical analyses from Fig. 9e to g using a Wilcoxon-Mann-Whitney test of 2^−ΔΔ^*^CT^* (ratio gene/housekeeping gene ZIKV versus CT). (b) Indirect IF studies were used to label blood vessels (IB4, green), ZO-1 (magenta), and nuclei (cyan). Bars, 10 μm. (c) Indirect IF studies were used to label blood vessels (IB4, green), claudin-5 (magenta), and nuclei (cyan). Bars, 10 μm. Download FIG S8, TIF file, 0.6 MB. Copyright © 2020 Clé et al. 2020 Clé et al. This content is distributed under the terms of the Creative Commons Attribution 4.0 International license.

**FIG 10:**
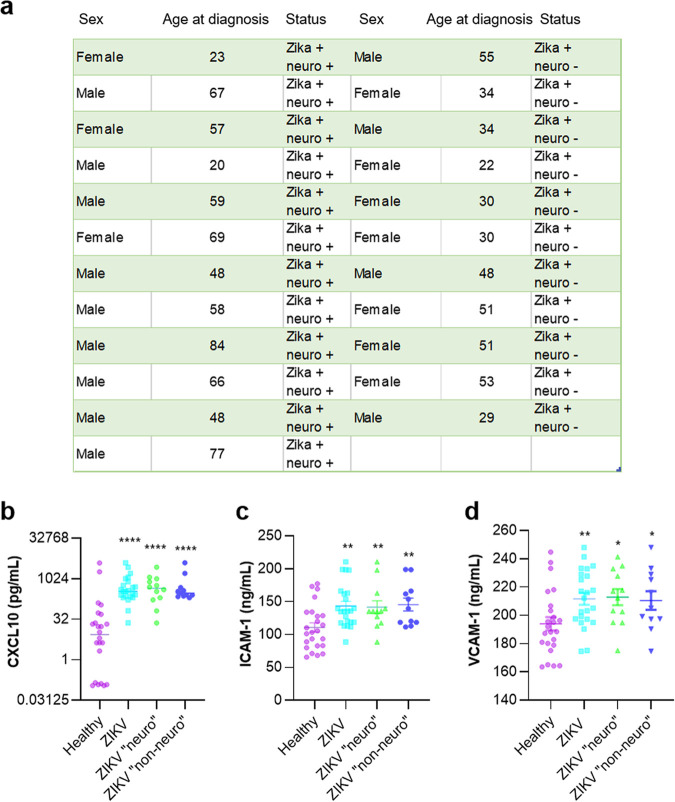
sCAMs are increased in the plasma samples from ZIKV^+^ patients. (a) ZIKV-infected patients from the CARBO cohorts. (b) CXCL10 levels in healthy blood donors and ZIKV^+^ patients were measured by ELISAs. Results are expressed as means ± SEMs (24 healthy, 24 ZIKV^+^ plasma samples [12 neuro and 12 non-neuro]) and analyzed using a Wilcoxon-Mann-Whitney test. ****, *P* < 0.0001 compared to healthy plasma. (c and d) Soluble ICAM-1 and VCAM-1 levels in healthy blood donors and ZIKV^+^ patients were measured by ELISAs. Results are expressed means ± SEMs (24 healthy, 24 ZIKV^+^ plasma samples [12 neuro and 12 non-neuro]) and analyzed using a Wilcoxon-Mann-Whitney test. *, *P* < 0.05; **, *P* < 0.01; ****, *P* < 0.0001 compared to healthy plasma samples.

Finally, we monitored sCAM levels in plasma samples from healthy blood donors and ZIKV^+^ symptomatic patients from the 2016 epidemic in the French West Indies. These patients displayed or not neurological symptoms upon hospital arrival (neuro and non-neuro, respectively) (Fig. 10a). We therefore performed ELISAs to measure CXCL10, ICAM-1, and VCAM-1 in these plasma samples and showed that, as previously described in ZIKV^+^ patients (39), the CXCL10 concentration was significantly higher in ZIKV^+^ patients, independently of their neurological status, as no statistically significative differences were found in levels between patients displaying neurological impairments and patients who did not (Fig. 10b). Levels of soluble ICAM-1 and VCAM-1 were also statistically modulated in ZIKV^+^ patients, also independently of their neurological status (Fig. 10c and d).

These sets of observations suggest that CAMs are modulated *in vivo* and in humans during neuroinfection and could participate/exacerbate neuroinflammatory mechanisms triggered by ZIKV.

## DISCUSSION

In this study, we showed that an *in vitro* multicellular human BBB model is permissive to direct ZIKV infection and replication, with partial impairment of its permeability during the course of infection. Moreover, pericytes and astrocytes, key components of the NVU and modulators of neuroinflammatory mechanisms, can also be targeted by ZIKV and allow viral replication. Interestingly, albeit viral replication was not deleterious, ZIKV in hBLECs, pericytes, and astrocytes led to the upregulation of some inflammatory cytokines (i.e., IL-6 and IL-8) and some chemokines involved in immune cell recruitment (i.e., CCL5 and CXCL10). An RT-qPCR array of genes involved in general endothelium homeostasis revealed that in hBLECs, ZIKV also led to the modulation of several genes involved in BBB physiology, such as CAMs and TJ proteins, arguing that the BBB could be perturbed (perhaps locally) during the course of infection. Modulation of CAM levels was responsible for an increased leukocyte recruitment/binding to ZIKV-infected BBB and could contribute to general immune cell CNS infiltration and inflammation-associated pathology. These observations were correlated by results in mouse models and, importantly, in the plasma of ZIKV^+^ patients.

Arboviruses, and particularly some flaviviruses, are known to interact with, and cross, the BBB using different mechanisms such as infected immune cell transcytosis, also known as the Trojan horse pathway, or by direct hBLEC infection and release into the parenchyma (17). For instance, WNV can reach the CNS by infecting monocytes, dendritic cells, or macrophages (40, 41). ZIKV has been also suggested to use this mechanism (42–44), and here we show that some CD45^+^ cells are recruited to cells lining blood vessels and display ZIKV antigen staining (Fig. 9d). The effect on BBB homeostasis of infected leukocytes is, however, still unclear. Direct infection of hBLECs by using various cell lines and *in vitro* models has been described for WNV, dengue virus (DENV), JEV, and recently, ZIKV (17, 21, 37, 45, 46). Recent studies and our results suggest that ZIKV can directly infect BBB cells but may not have a strong deleterious effect on BBB integrity and that viral particles could be released basolaterally and reach the CNS (21). Other arboviruses such as WNV and JEV lead to endothelium integrity impairment and inflammatory molecule production that will disrupt BBB integrity and further allow virus CNS access (19, 47). Among these cytokines, tumor necrosis factor alpha (TNF-α), IL-1β, transforming growth factor beta (TGF-β), and IL-6 have been shown to modulate BBB permeability by several mechanisms, including downregulation or relocalization of junction proteins such as occludin and ZO-1 (48, 49). Modulation of TJ and AJ protein expression in arbovirus infection may increase viral and immune cell CNS access by paracellular pathways (50).

In adults, ZIKV infection can be associated with peripheral neuropathology (Guillain-Barré syndrome) but also, in some cases, with CNS disorders such as encephalitis and encephalomyelitis (16, 51, 52). The characterization of the molecular and cellular mechanisms governing ZIKV CNS access are therefore particularly important. A few studies addressed this subject using *in vitro* BBB models. However, modeling the human BBB is still rather challenging, and in many cases, brain vascular immortalized cell lines are used, which are far from reproducing *in vivo* physiological properties, in particular, in terms of permeability (30, 31, 53). Promising and innovative *in vitro* models are starting to emerge, using stem cell- and IPSC-derived human BECs, to recreate 3D or flow properties (30, 53). Similarly to our observations in the present hBLEC model, a study using IPSC-derived BECs (22), as well as work using human brain microvascular endothelial cells (HBMECs) (20) (cells isolated from pediatric and adult patients and kept in culture for several passages [54]) show efficient direct infection and release from both apical and basolateral sides without disruption of the endothelium integrity. However, these models are not completely pertinent, as the NVU is a complex multicellular system. In our system, hBLECs are in contact with factors produced by pericytes that allow the regulation of endothelium homeostasis and may participate/exacerbate inflammatory events occurring in these cells. Even though we detected efficient ZIKV replication of two African and Asian ZIKV strains, which both underwent a limited number of amplification passages (33), we nonetheless did not observe a strong impairment of the BBB integrity. However, albeit the permeability coefficient (Pe) indicated an impermeable endothelium, ZIKV infection led to a Pe increase (i.e., an increase in permeability) of a very small lipophilic marker (i.e., LY, 442.3 Da) and a decrease of TEER, which could suggest that the endothelium integrity was partially perturbed. This was then confirmed by a visualization of actin network reorganization, with a potential subtle downregulation of proteins regulating BBB integrity such as TJ proteins (occludin, ZO-1, and claudin-5), PECAM-1, and transporters (e.g., PgP). This set of observations could imply that the general BBB homeostasis and function may indeed be impaired by ZIKV.

In this study, we found that the chemoattractive molecules CXCL10, CCL5, and CCL2 were potently released by the apical compartment upon ZIKV hBLEC infection, suggesting that circulating leukocytes could be attracted to the infected BBB. CXCL10 is emerging as a key inflammatory molecule expressed during neuroinflammatory processes such as autoimmune disorders (e.g., multiple sclerosis [55] or encephalitis [47, 56]). It is involved in the recruitment of T cells to the BBB and was proposed to favor inflammatory cell recruitment into the CNS following rabies virus infection (56). Interestingly, it was also detected in plasma samples from ZIKV^+^ patients (our results and those in reference 39). Moreover, CCL5 and CCL2 are known mediators of leukocyte recruitment to the BBB (57, 58), thus suggesting that the local environment in ZIKV-infected BBB could favor immune cell recruitment/migration and access to the CNS. Besides the upregulation of chemoattractants, one of the key observations of this study was the marked increase in CAM expression, namely, ICAM-1, VCAM-1, and E-selectin, in ZIKV-infected hBLECs. Using different approaches, we showed that this upregulation occurred through the endothelium and was not only restricted to infected cells. CAMs play crucial roles in BBB homeostasis, one of which is to mediate immune cell (e.g., leukocytes) capture, docking, and transmigration (59). This is an important step, as it is crucial for CNS immune surveillance, particularly in an inflammatory state (e.g., encephalitis and meningoencephalitis). CAMs present at the surfaces of endothelial cells are involved in numerous steps of leukocyte diapedesis, namely, capture/rolling (dependent on selectins), arrest (dependent on ICAM-1/VCAM-1), crawling, and diapedesis *per se* (60–62). Leukocytes can cross the BBB using para- or transcellular transmigration, without TJ and barrier disruption (59). This has been well documented in experimental autoimmune encephalomyelitis (EAE) models (63). In EAE, as well as in multiple sclerosis (MS), ICAM-1, VCAM-1, and ALCAM are upregulated (59). Noteworthy, the cell surface level of ICAM-1 was shown to be directly proportional to T cell diapedesis (64). Besides their “physical role” as a docking factor, CAMs display intracellular functions that will support diapedesis: for instance, intracellular signaling is associated with ICAM-1 and VCAM-1 in brain vascular endothelial cells. The small GTPase Rho, a potent regulator of the actin cytoskeleton, can be activated upon ICAM-1 engagement by leukocytes, leading to actin rearrangement and TJ and AJ protein redistribution, allowing diapedesis (65). Here, we show that both monocytes and CD4^+^ T cells (LyT) showed an increase in binding to hBLECs under ZIKV infection conditions. Moreover, LyT displayed a strong change in morphology, suggestive of the first step of diapedesis. In this context, CAMs were found to be necessary for both binding and “spreading,” as a cocktail of blocking antibodies strongly reduced both the total number and the size of bound LyT. Interestingly, a recent study reported that ZIKV-infected monocytes also displayed upregulation of CAMs such as ICAM-2 and V-cadherin and increased adhesion properties (66).

Upregulation of CAMs was also observed in the case of WNV CNS infection both *in vivo* (in animal models) and *in vitro* in human brain microvascular endothelial cells (HBMVECs; a brain endothelial cell line) and was proposed to be responsible for leukocyte diapedesis (37, 67). Similarly, upregulation of VCAM-1 in microvascular endothelial cell lines infected with dengue virus (DENV) was reported (45), whereas supernatant from DENV-infected monocytes led to the increase in ICAM-1, VCAM-1, and E-selectin levels in human microvascular endothelial cells (HMVECs) (68). Here, we also found an increase in the concentration of soluble forms of ICAM-1 and VCAM-1 in the apical supernatant of ZIKV-infected hBLECs as well as in the plasma samples from ZIKV^+^ patients. In the context of CAMs, circulation of soluble extracellular domains is often associated with inflammatory states. Their serum levels are increased in cardiovascular disorders such as arteriosclerosis and in some forms of cancer (69). Moreover, they can be found in acute disseminated encephalomyelitis, which is a postinfectious inflammatory disease, and in the serum and cerebrospinal fluid (CSF) of patients with multiple sclerosis, where their level correlates with disease severity (70). Similar observations were made in patients suffering from Parkinson’s disease (71), highlighting the potential relation between sCAM levels in biological fluids and inflammation. Interestingly, an increase in soluble VCAM-1 has been detected in severe DENV infections and was proposed to represent a marker to monitor disease severity (72). To clinicians, the use and characterization of serum biomarkers is pertinent not only to predict disease severity but also potential neurological impairment (e.g., by detecting neuronal factors resulting from increased BBB permeability). In ZIKV^+^ patients, however, we did not detect differences in sCAM levels depending of the neurological status at the time point tested (still in the viremic phase). We could therefore not conclude that levels of sCAMs correlate with ZIKV CNS targeting; therefore, levels could be predictive of CNS impairment during infection. It is possible that the general inflammatory response triggered by ZIKV in humans precludes specific detection in the plasma of ubiquitous molecules that are also modulated during CNS invasion or that differences appear later in the disease progression. One could also speculate that in some patients, ZIKV could reach the CNS without leading to strong and detectable neurological impairments. Nonetheless, adult CNS targeting during ZIKV infection could be a combination of strain-dependent virulence, inflammatory environment, and individual genetic background.

Moreover, we showed that human pericytes were infected by ZIKV, also leading to the production of cytokines and chemokines. Due to their critical role in the NVU in BBB endothelial cell biology, modulation of pericyte homeostasis can have direct effects on barrier integrity (29). Moreover, pericytes have also been shown to directly regulate leukocyte diapedesis once they crossed the endothelial cell layer (73). Very few studies report on pericyte viral infection and their consequences: HIV was shown to efficiently infect pericytes and, in turn, trigger cellular dysfunction and inflammatory responses, which in some cases, may affect BBB integrity (74). Similarly, JEV can also infect brain pericytes, which, through the action of inflammatory molecules, will destabilize the brain endothelial barrier (75). Here, we detected efficient, albeit reduced, viral replication. This translated in the modulation of several genes involved in immune response, in particular, some chemokines such as CXCL10 and CCL5 and inflammatory cytokines such as IL-6 and IL-8. One could therefore draw the hypothesis that ZIKV release from the BBB basolateral compartment and further pericyte infection could favor the production of a local inflammatory environment. Interestingly, in ZIKV-infected retinal pericytes were also shown to be infected by ZIKV (76). One limitation in our study, however, is the use of bovine pericytes in the bi- and triple culture models. It will be important to use human pericytes in these models, as inflammatory responses may be different in infected human versus bovine pericytes. We also observed that astrocytes potentiate the inflammatory response in infected BBB, as levels of apically secreted CCL5 appeared to be more important when hBLECs were cultured with astrocytes and pericytes. The infection of astrocytes by ZIKV in close proximity to the BBB, concomitantly with pericytes, could strengthen the release of cytokines and chemokines and the modulation of BBB integrity as well as the recruitment of leukocytes from the blood.

A parallel between some of these observations and our previous work on the blood-retinal barrier, in particular, the retinal pigment epithelium (RPE) (32), is pertinent to draw here. Indeed, we and others have described the infection of several cell types of the blood-retinal barriers, which can explain the ocular disorders associated with ZIKV infection (77). Albeit some similar mechanisms in the induction of chemokines and some inflammatory cytokines were reported here (i.e., upregulation of *CCL5*, *CXCL10*, *IFNB*, *IL6*, etc.), it is noteworthy that ZIKV AF and ZIKV AS had a much stronger effect on the RPE integrity and homeostasis. Indeed, at stages of infections where the BBB was not perturbed, RPE impermeability was completely abolished, and electron microcopy studies revealed the (almost complete) epithelium disruption (32). Moreover, ZO-1 staining was nearly abolished in ZIKV-infected RPE, altogether demonstrating that cell-cell adhesion was strongly impaired. Here, on the contrary, the endothelium organization and integrity were only subtly affected. The balance in the production of cytokines and chemokines seems to be in line with the modest effect on barrier integrity, as neither IL-1β or TNF-α and very limited amounts of IFN-γ were produced, cytokines previously shown to mediate BBB loss of integrity triggered by WNV and JEV (19, 47). We report here nonetheless that BBB permeability was partially perturbed by direct ZIKV infection both *in vitro* and *in vivo*. A recent study also suggests that ZIKV can slightly modulate BBB integrity (23) and could be consistent with a local effect during infection, allowing virus entry and recruitment of immune cells. This was also illustrated during *in vivo* infection by JEV, where increased BBB permeability was observed in the cerebrum but not in the cerebellum, suggesting that differential alterations in the barrier properties of cerebrum and cerebellum microvascular endothelial cells could likely occur depending on the inflammatory stimuli involved (78).

It is noteworthy, however, that the use of *Ifnar^−/−^* mice has a clear limitation when parallels to ZIKV human (neuro)pathology need to be drawn (79). In particular, since IFN pathways are involved in BBB integrity regulation, their modulation in mice could affect BBB homeostasis *per se*, since type I IFN can stabilize the BBB and modulate the expression of TNF-α and IL-1β, known to perturb BBB integrity (2). For instance, *Ifnar^−/−^* mice subjected to WNV infection displayed an increased impairment of BBB permeability compared to that of WNV-infected wild-type (WT) animals (80). Regarding ZIKV and animal models, WT mice do not display strong sensibility to infection, due to the absence of degradation of murine STAT2 and a subsequent clearance of the virus (79). Therefore, to study ZIKV, mice deficient in IFN signaling are used a majority of the time, with the limitations described above. Interestingly, one study, however, demonstrated strain-dependent BBB impairment in immunocompetent mice (23). The use of nonhuman primate models showed that ZIKV reached the CNS and could provide complementary approaches to better study the effect of ZIKV on BBB homeostasis *in vivo* (79). In this light, compromised blood-brain barrier (loss of ZO-1) was observed in new world monkey models subjected to ZIKV infection (81).

Finally, one could speculate on the differential neurotropism and local BBB inflammation/perturbation between ZIKV AF and ZIKV AS, as we observed higher replication and, in some cases, particularly in pericytes and astrocytes, stronger inflammatory responses triggered following infection *in vitro* by ZIKV AF. Numerous studies now point toward differences between African and Asian strains, with a generally higher virulence and cell toxicity associated with ZIKV of African origins (12, 82). It is not clear, however, whether differences in CNS access in adults exist between the different strains. One study reported differences between ZIKV strains in BBB modulation (23), and neural cell attachment and neurotoxicity dependent on ZIKV infection were proposed to be dependent on the prM-E protein (83). Here, we show that *in vitro*, ZIKV AF displayed a stronger apical and basolateral viral release, and in the mouse CNS, ZIKV AF replicated more efficiently and led to stronger upregulation of some inflammatory and adhesion molecules than ZIKV AS, suggesting that African strains may have better access to the adult CNS.

Altogether, our observations would favor a hypothesis where ZIKV direct infection of brain vascular endothelial cells would allow viral replication and possible delivery into the parenchyma, a mechanism that, in combination with the Trojan horse pathway, would favor ZIKV access to the CNS. Modulation of surface proteins such as CAM and of junction actor modulators, as well as secretion of some chemokines and inflammatory molecules, would help recruit leukocytes, which would engage in diapedesis and further infiltrate the CNS, favoring neuroinflammation. Pericytes, which are now well described as mediators of neuroinflammation, as well as astrocytes, could be infected and support this inflammatory state, possibly in local areas of the BBB.

## MATERIALS AND METHODS

### Materials.

The antibodies used in this study were as follows: mouse anti-pan-flavivirus (clone 4G2, MAB10216; Millipore), rabbit anti-ZO1 (617300; Invitrogen), and rabbit anti-glyceraldehyde-3-phosphate dehydrogenase (GAPDH) (G9545; Sigma-Aldrich) antibodies, rabbit anti-VCAM-1 (clone EPR 16589; Abcam) and blocking antibodies (BBA5; R&D Systems), rabbit anti-ICAM-1 (clone 9HCLC; Abcam) and blocking antibodies (BBA3-200; R&D Systems), and mouse anti-hE-selectin (BBIG-E1; R&D Systems), rabbit anti-PDGF receptor beta (ab32570; Abcam), mouse anti-dsRNA (J2; Scicons), rabbit anti-ZIKV Env (GeneTex), rat anti-CD45 (14-0451; Bioscience), rabbit anti-CD3 (A0452; Agilent), and rabbit anti-claudin-5 (341600; Invitrogen) antibodies. Hoechst was purchased from Merck, and isolectin B4 was purchased from Vector Laboratories.

Vero cells (ATCC, USA) were grown in Dulbecco’s modified Eagle’s medium (DMEM) containing 10% or 2% heat-inactivated fetal bovine serum (HI-FBS), 100 μg/ml streptomycin, 100 U penicillin, 2 mM l-glutamine, 1% sodium bicarbonate, and 1% HEPES buffer (all from Pan Biotech). C636 cells were grown in RPMI medium containing 10% or 2% HI-FBS, 100 μg/ml streptomycin, 100 U penicillin, without l-glutamine but with 2.0 g/liter NaHCO_3_ (all from Pan Biotech). Human pericytes and astrocytes were purchased from ScienCell and cultured according to the manufacturer’s instructions. Cells were cultured on poly-d-lysine-coated plates and were used between passage 2 and 4.

### Virus strains.

We used previously published ZIKV AF and AS strains (32, 33). Briefly, H/PF/2013 ZIKV of Asian lineage (French Polynesia, 2013) and ArB41644 ZIKV of African lineage (Bangui, Central African Republic, 1989) were produced and provided by the National Reference Center for arboviruses at fewer than 5 passages on Vero cells. Viral titers were determined by the 50% tissue culture infective dose (TCID_50_), which was calculated using the Spearman-Kärber method (84), and were expressed as TCID_50_ per milliliter.

### *In vitro* human BBB models.

This model requires the collection of human umbilical cord blood, for which infants’ parents signed an informed consent form, in compliance with French legislation. The protocol is approved by the French Ministry of Higher Education and Research (CODECOH number [no.] DC2011-1321). All experiments were carried out in accordance with the approved protocol. Hematopoietic stem cells positive for the CD34 marker were isolated and purified from umbilical cord blood and then differentiated into endothelial cells as previously described (85). Then, the CD34^+^ blood cord-derived endothelial cells (CD34^+^-EC) were seeded on Matrigel-coated Transwell filters (Costar, 0.4 μm) on top of bovine pericytes in 12-well plates as previously described (25). This model is then named human brain-like endothelial cells (hBLECs) and reproduces the main features of the human BBB (25, 28). Briefly, once plated, CD34^+^-EC and pericytes were cultured for 5 to 6 days, with medium changes every 2 days. Then, endothelial permeability (Pe) was tested by measuring Lucifer yellow (LY) (20 μM; Life Technologies) transendothelial crossing using established protocols (25, 27). Pe was measured after 1 h of LY transport by calculating the concentration-independent parameter as previously published (27). The fluorescence detection was performed using a Tecan SPARK 10M apparatus with excitation/emission wavelength (nm) settings of 432/538 nm. Infection experiments were carried when the barrier was impermeable (i.e., with a Pe of ≤1 × 10^−3 ^cm/min). ZIKV was added at the correct MOI in 200 μl of complete endothelial medium (ECM) for 2 h on an orbital shaker. Three hundred microliters of ECM was then added and the inoculum removed. Infections were then carried for 4, 7, or 10 days. For each experiment, triplicates were used.

The TEER was measured using the Epithelial Volt/Ohm Meter EVOM2 (World Precision Instruments, Hertfordshire, UK) according to the manufacturer’s instructions. Briefly, electrodes were sterilized in 70% ethanol for 5 min, rinsed and equilibrated in media, and then placed in the compartmentalized chambers with the longer electrode vertically touching the bottom of the dish in the lower chamber and the shorter electrode in the upper chamber without touching the cell layer. TEER was recorded once the value stabilized, approximately 5 s after placing the electrode. To calculate the final TEER values (Ohms·cm^2^), the background measurement of a Matrigel-coated insert without cells was subtracted from the reading and the value multiplied by the growth surface area.

For triple-culture experiments, pericytes were seeded on the day of plating onto the gelatin-coated bottom of the Transwell filter (Costar, 0.4 μm), and 3 h later, CD34^+^-EC were seeded on the Matrigel-coated top part of the filter. Coculture was allowed for 6 days, and Pe was tested via the LY protocol. The following day, human primary astrocytes were plated on poly-l-lysine-coated 12-well plates, and filters were transferred according to published protocol (35). In this set up, there is no contact between the different cell types. Infection with ZIKV was concomitantly performed.

### Leukocyte adhesion assays.

T cells and monocytes were purified according to established protocols and maintained for 7 days with the cytokine IL-2 (T cells CD4^+^) or used 12 to 18 h postisolation (monocytes). Buffy coats from healthy donors were obtained from the Etablissement Français du Sang (EFS, Montpellier, France). After Ficoll gradient on peripheral blood mononuclear cells (PBMCs), monocytes were isolated using CD14 MicroBeads (Miltenyi Biotec); purity was >95% CD14^+^. Adhesion assays were conducted as previously published (27). Briefly, immune cells (LyT or monocytes) were labeled using the CFSE (carboxyfluorescein succinimidyl ester) probe according to the manufacturer’s instructions (Life Technology) and incubated for 30 min on hBLECs in ECM. Blocking experiments were performed by incubating with a cocktail of neutralizing antibodies against ICAM-1, VCAM-1, and E-selectin (10, 20, and 25 μg/ml, respectively; R&D systems [see above]) for 1 h prior to the adhesion assay. Cells were then gently rinsed with PBS, filters were fixed with 4% paraformaldehyde (PFA) for 15 min at room temperature (RT), and indirect immunofluorescence assessment was then performed. The “spread” phenotype was determined when the general cell shape was not circular and some cell ruffles or filopodium-like structures were observed. This descriptive method was further confirmed by analyzing the cell diameter.

### Mouse experiments and ethics statement.

Pathogen-free *Ifnar^−/−^* mice (86) kindly provided by Giles Uzé were bred in the animal facilities at CECEMA (Centre d'Elevage et de Conditionnement Expérimental des Modèles Animaux). Groups of 6- to 8-week-old *Ifnar^−/−^* mice were infected with ZIKVPF13 or ZIKVArB41644, as described previously (32). Briefly, groups of 6- to 8-week-old mice were inoculated via an intraperitoneal route with 10^4^ TCID_50_/ml of ZIKV AS or ZIKV AF. At 7 dpi, mock- and ZIKV-infected mice were intravenously (i.v.) injected with 0.5% Evans blue solution (EB; 200 μl per mouse) and euthanized after PBS intracardiac perfusion following a lethal dose of pentobarbital (Sigma-Aldrich, Darmstadt, Germany). Brains were either fixed in 4% PFA and cut using a microtome (3-μm sections) at the RHEM facilities (Montpellier) or snap-frozen. Mice were bred and maintained according to the French Ministry of Agriculture and European institutional guidelines (appendix A STE no. 123). Experiments were performed according to national regulations, and this study was specifically approved (approval no. 6773-201609161356607) by the regional ethics committee of Languedoc-Roussillon (Comité Régional d'Ethique sur l'Expérimentation Animale- Languedoc-Roussillon), France.

### Human samples (CARBO cohort).

CARBO (Cohort Arbovirosis) is a descriptive and prognostic study of arbovirosis in France, based on a hospital cohort of children and adults with suspected arbovirosis. The main objective of the study was to define the predictive factors of severe arbovirosis. This study is registered at clinicaltrials.gov (NCT01099852). Ethical clearance was obtained from the French National Agency for the Safety of Medicines and Health Products (ANSM) (no. IDRCB 2010-A00282-37) and by the committee for the protection of individuals. Written and signed informed consent of all subjects was obtained. For this study, patient inclusion was conducted from December 2015 to December 2016. Inclusion criteria were suspicion of ZIKV infection, ability to sign informed consent, blood or urine sample positive for ZIKV RNA by reverse transcription-PCR (RealStar Zika Virus RT-PCR kit 1.0; Altona Diagnostics, Hamburg, Germany), and onset of symptoms of ≤21 days. Data were collected at the initial visit and, if possible, at day 3 after the onset of symptoms, between days 5 and 7, between days 8 and 10, at day 21, and at weeks 6 and 12, including sociodemographic data, comorbidities, and clinical characteristics. Neurologic manifestations were classified as either involving the peripheral nervous system (PNS) only, CNS only, or involving both PNS and CNS (mixed disorders). A diagnosis of Guillain-Barré syndrome was made using international Brighton criteria. Patients with encephalitis or acute myelitis were diagnosed according to consensus criteria.

### Statistics.

For all quantitative analyses, a minimum of three independent experiments were performed. Unpaired *t* tests (i.e., Wilcoxon-Mann-Whitney) were performed to analyze unpaired data.
