# Supplementary material for: Zika Virus Infection Promotes Local Inflammation, Cell Adhesion Molecule Upregulation, and Leukocyte Recruitment at the Blood-Brain Barrier
Source: mBio. 2020 Aug 4;11(4):e01183-20. doi: 10.1128/mBio.01183-20 (PMC7407083; doi:10.1128/mBio.01183-20)
Supplement: TEXT S1 [file mBio.01183-20-s0001.docx]

**Supplemental Material and Methods**

**Immunoblotting**

Cells were rinsed with PBS and scraped in TNE (Tris-NaCl-EDTA with 0.1% NPA40) buffer, then centrifuged to remove cellular debris. The samples were denatured 5 min at 95 °C and loaded on a TGX Stain Free gel (Biorad). The separated proteins were transferred to a PVDF membrane using a Trans-Blot® Turbo™ System (Biorad). Membranes were saturated for 1 h at RT in 0.5% Tween-PBS in 5% skim milk (blocking solution), incubated with 1:500 dilution of anti-ICAM-1, or anti-VCAM-1 (Abcam), or with 1:1000 dilution of anti-GAPDH overnight at 4°C. Membranes were washed in 0.5%Tween-PBS, and incubated with 1:5000 dilution of horseradish peroxidase (HRP)-conjugated goat antibody against mouse or rabbit (Millipore). Detection was performed using ECL (Biorad) and a Biorad Chemidoc system.

**RT-qPCR assays**

ZIKV-or mock-infected hBLEC grown on transwell or pericytes grown on 6-well plates were harvested in RLT buffer (Qiagen) and total RNA was extracted using RNeasy® mini-kit (Qiagen). Complementary DNA was synthesized using Omniscript® reverse transcriptase (Life Technologies). RT_2_ Profiler PCR arrays for Human Endothelial cell Biology (BBB, #PAHS-015Z, 96 well format, Qiagen) or for Human Innate and Adaptive Immune responses (Pericytes, #PAHS-052Z, 96 well format, Qiagen) were used for real-time quantitative PCR (RT-qPCR) analyses, were performed using the LC480 real time PCR instrument (Roche) and the Light Cycler 480 SYBR Green I master Mix (Roche). Volumes of mix, cDNA, RNAse-freewater, and cycling conditions were determined according to the manufacturer's instructions. Gene expression was normalized to that of the housekeeping gene HPRT. Genes without interpretable amplification curves were excluded from the analysis.

Organs were weighed, homogenized with zirconia beads in a Fastprep 24 apparatus (MP Biomedicals) and stored at −80°C. RNA was extracted using the RNeasy Mini Kit (Qiagen). Viral RNA levels were measured by one-step quantitative reverse transcriptase PCR (RT-qPCR) using primers against the NS5 gene on an ABI 7500 Fast Instrument using standard cycling conditions. Viral burden was expressed on a log10 scale as copies/μl after comparison with a standard curve.

**Indirect immunofluorescence assays**

hBLECs on Matrigel-coated cell culture inserts, and pericytes were infected or mock-treated. For indirect IF, cells were fixed with 4% PFA and permeabilized with 0.1% Triton X-100/PBS for 5 min at room temperature (RT), followed by a blocking step with 2% bovine serum albumin (BSA) and 10% horse serum for 1h at RT. Primary and secondary antibodies were diluted in blocking solution and incubated sequentially for 1h at RT. When indicated, cells were treated during the secondary antibody incubation with ActinGreen (ThermoFischer scientific). Hoechst (Thermofischer) nuclei counter stain was performed during the secondary antibody incubation. Samples were mounted with fluorescent mounting medium (Fluoroshield, Sigma) and imaged by confocal microscopy using the Zeiss SP85 confocal microscope, with 40x or 63x 1.4 NA Plan Apochromat oil-immersion objectives. Apoptosis quantification was performed by imaging and counting condensed nuclei counterstained with Hoechst.

**Immunohistology**

Murine samples (see below) were collected and fixed 24h in neutral buffered formalin 10%, dehydrated, and embedded in paraffin. Paraffin-embedded brains were cut into 3-µm-thick sections, mounted on slides, then dried at 37°C overnight. Following deparaffination with Discovery EZ Prep solution at 75°C for 24 minutes, antigen retrieval was performed using Discovery CC1 buffer for CD45 and CD3 staining. Endogenous peroxidase was blocked with Discovery Inhibitor CM for 8 minutes at 37°C. Slides were incubated after rinsing at 37°C for 60 minutes with primaries antibodies. Signal enhancement was performed using the Discovery DAB Goat OmniMap Kit for CD3e, Discovery DAB Rabbit OmniMap Kit as the secondary antibody (Thermo Scientific, 31219) and the Discovery DAB Rabbit OmniMap Kit for CD45 staining. For viral antigen immunostaining, antigen retrieval was performed for 4 minutes at 37°C with Protease 1 solution from Ventana which is an endopeptidase (alkaline protease) of the serine protease family. Endogenous peroxidase was blocked with Discovery Inhibitor CM for 8 minutes at 37°C. The slides were incubated after rinsing at 37°C for 32 minutes with a mouse anti-flavivirus group antigen monoclonal antibody (Millipore, MAB10216, 1∶800 in Dako antibody diluent with background reducing components). Signal enhancement was performed using Rabbit monoclonal to mouse IgG1 + IgG2a + IgG3 as the secondary antibody (Abcam, ab133469, 1/8000) and the Discovery DAB Rabbit HQ Kit. Slides were imaged with a Hamamatsu NanoZoomer 2.0-HT scanner and visualized with the NDP.view 1.2.47 software.

**ELISA (Enzyme-Linked Immunosorbent Assay) and Multiplex analyses**

ELISA assays for human CXCL10, IL6, CCL2, CCL5, VCAM-1 and ICAM-1 were performed in mock- and ZIKV-infected supernatants from hBLEC, human pericytes and plasma-EDTA from CARBO patients (see below) according to the manufacturer’s instructions (R&D systems). Reading were done on spectrophotometer (Thermofischer Scientifics). For quantification of IFN-ϒ, IFN-λ, IL-8 in mock- and ZIKV-infected supernatants from hBLEC we applied the bead-based multiplex assay LEGENDPlex (BioLegend) and analyzed by FACSCanto at 4 and 7 days post infection according to the manufactor’s instructions.
