# Supplementary figures and images for: Zika Virus Infection Promotes Local Inflammation, Cell Adhesion Molecule Upregulation, and Leukocyte Recruitment at the Blood-Brain Barrier
Source: mBio. 2020 Aug 4;11(4):e01183-20. doi: 10.1128/mBio.01183-20 (PMC7407083; doi:10.1128/mBio.01183-20)

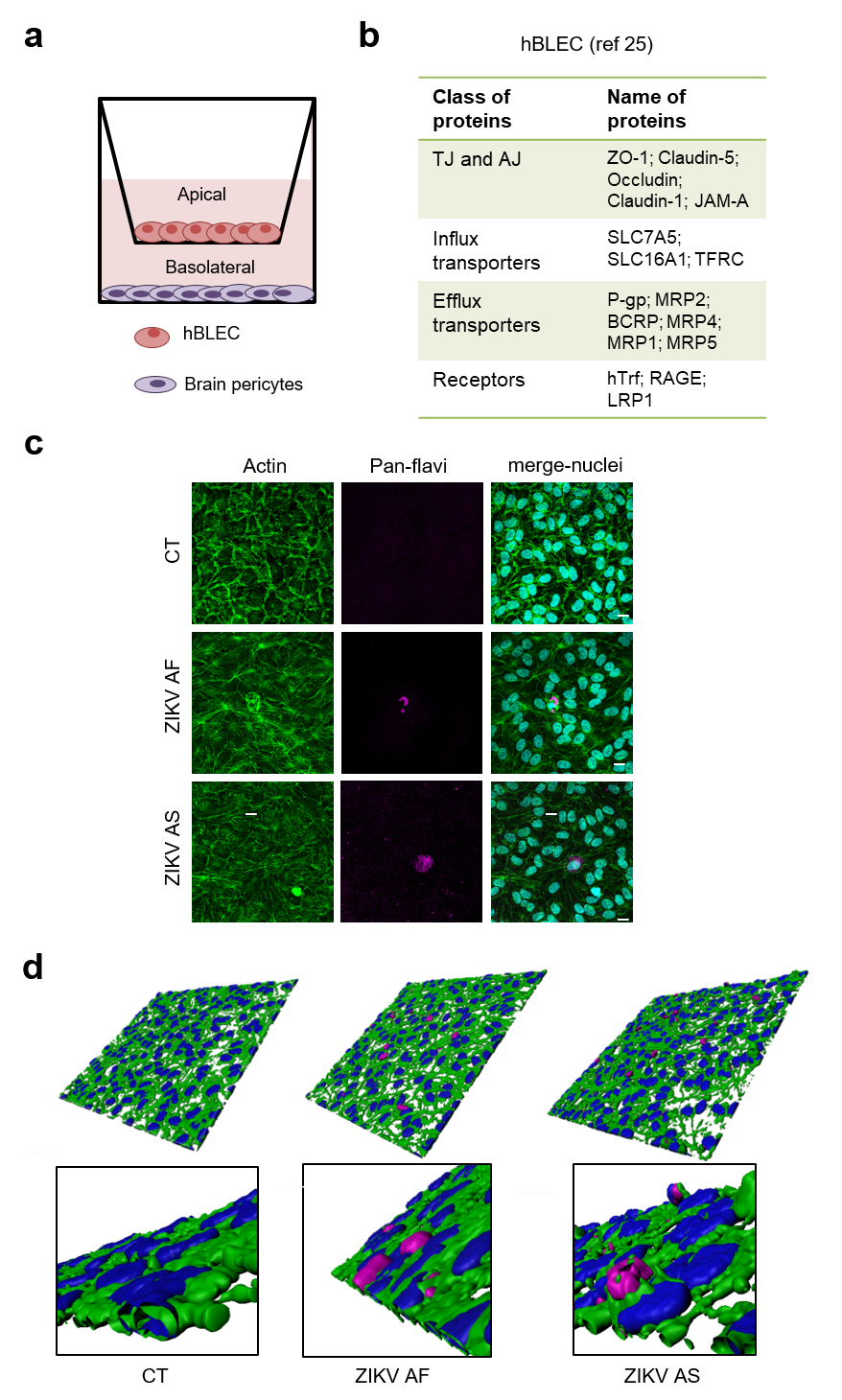

Supplement: FIG S1 [file mBio.01183-20-sf001.tif]

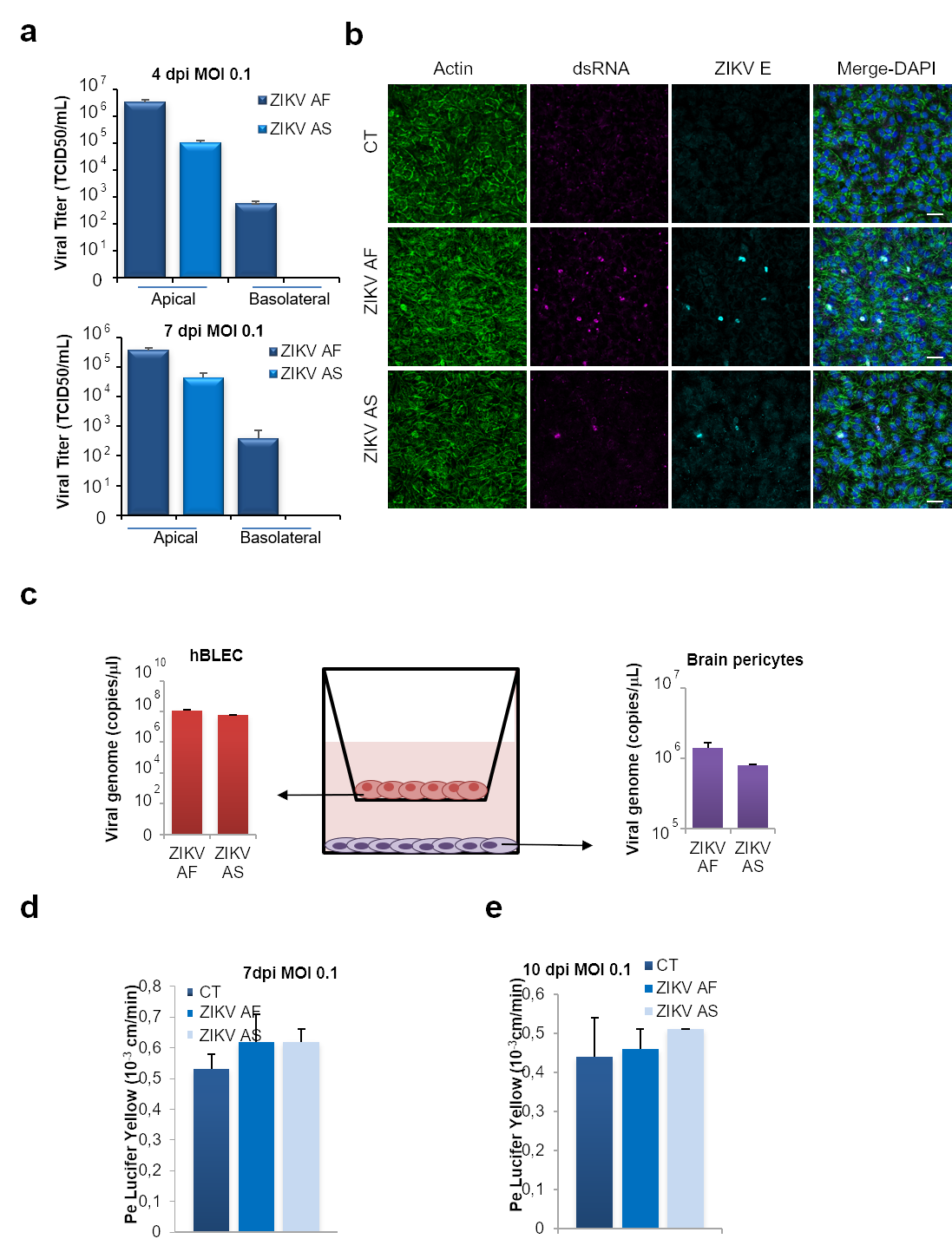

Supplement: FIG S2 [file mBio.01183-20-sf002.tif]

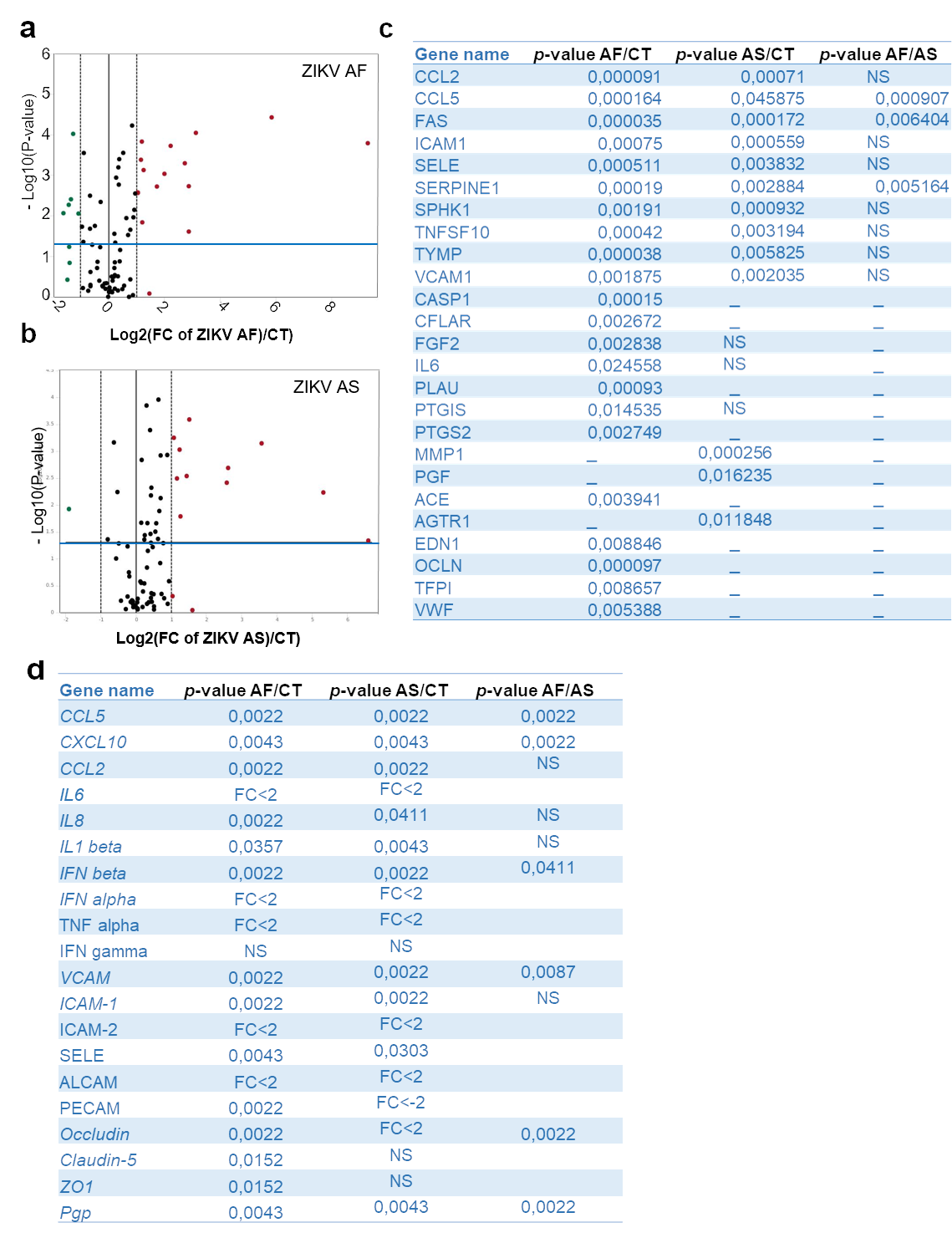

Supplement: FIG S3 [file mBio.01183-20-sf003.tif]

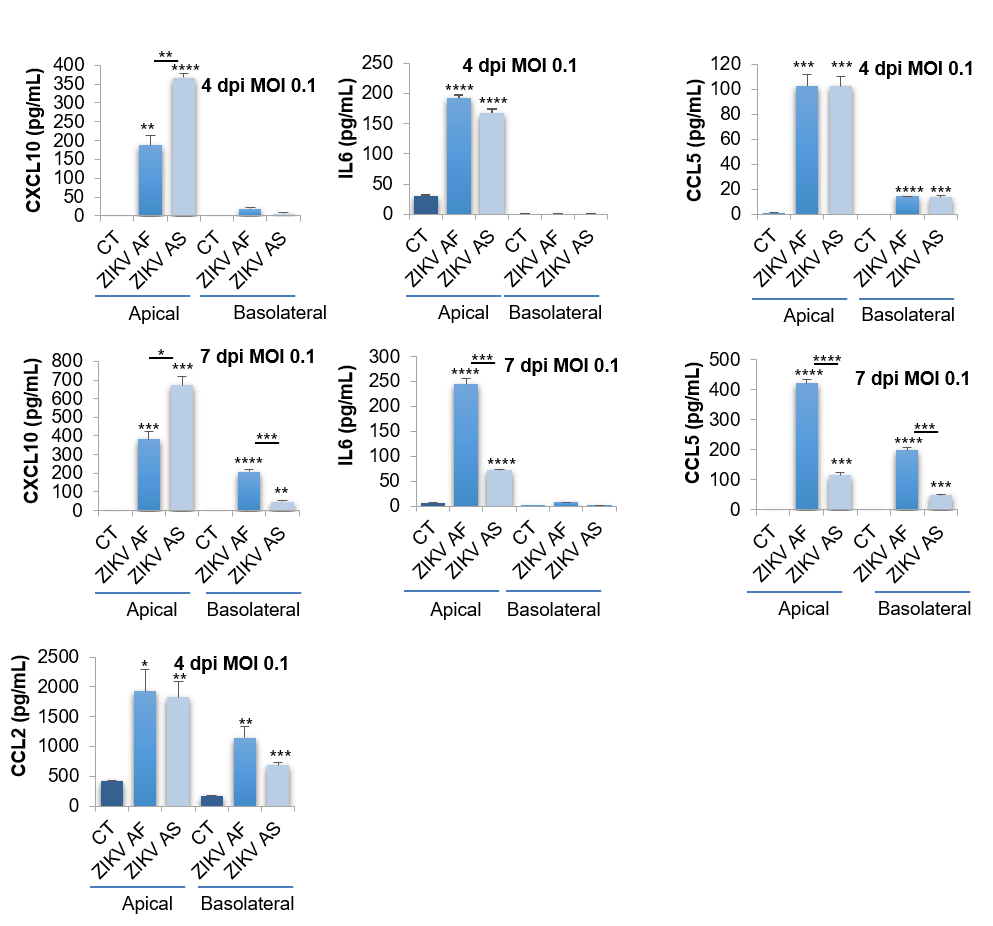

Supplement: FIG S4 [file mBio.01183-20-sf004.tif]

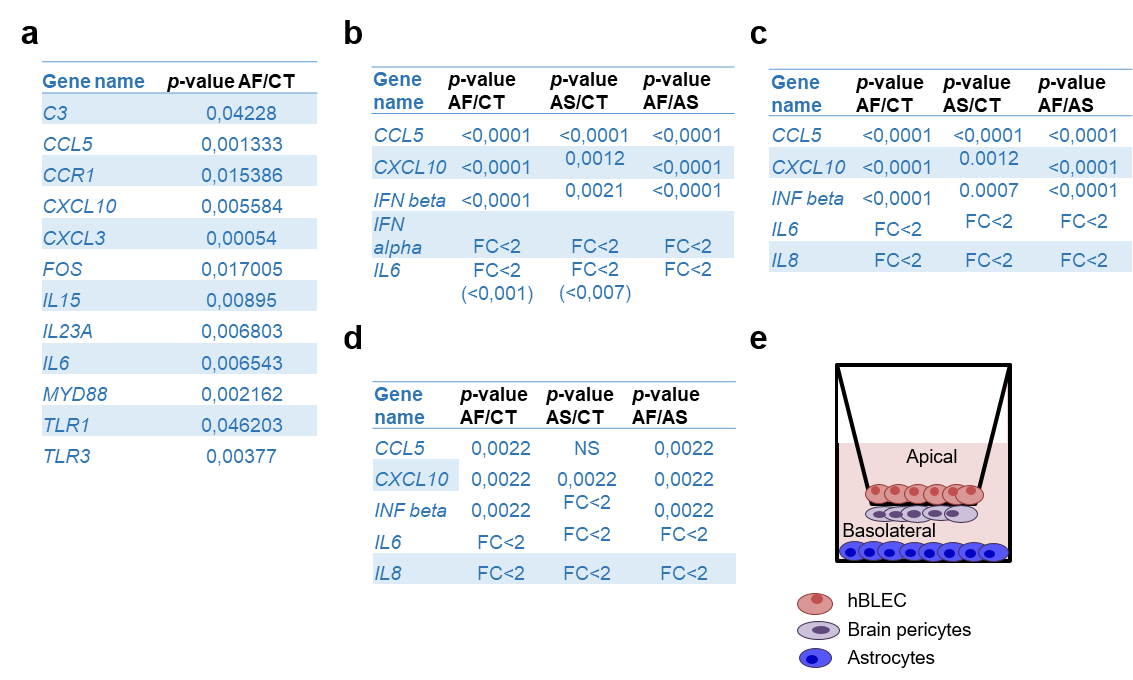

Supplement: FIG S5 [file mBio.01183-20-sf005.tif]

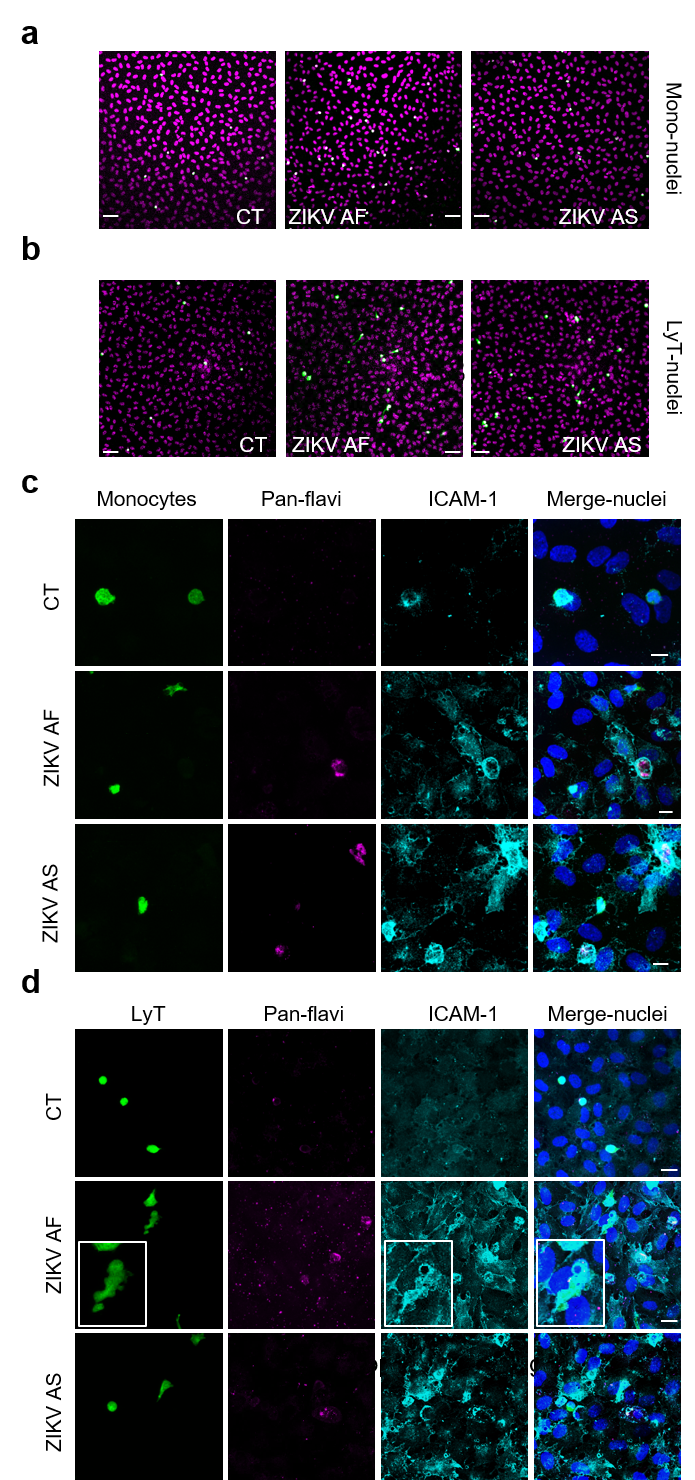

Supplement: FIG S6 [file mBio.01183-20-sf006.tif]

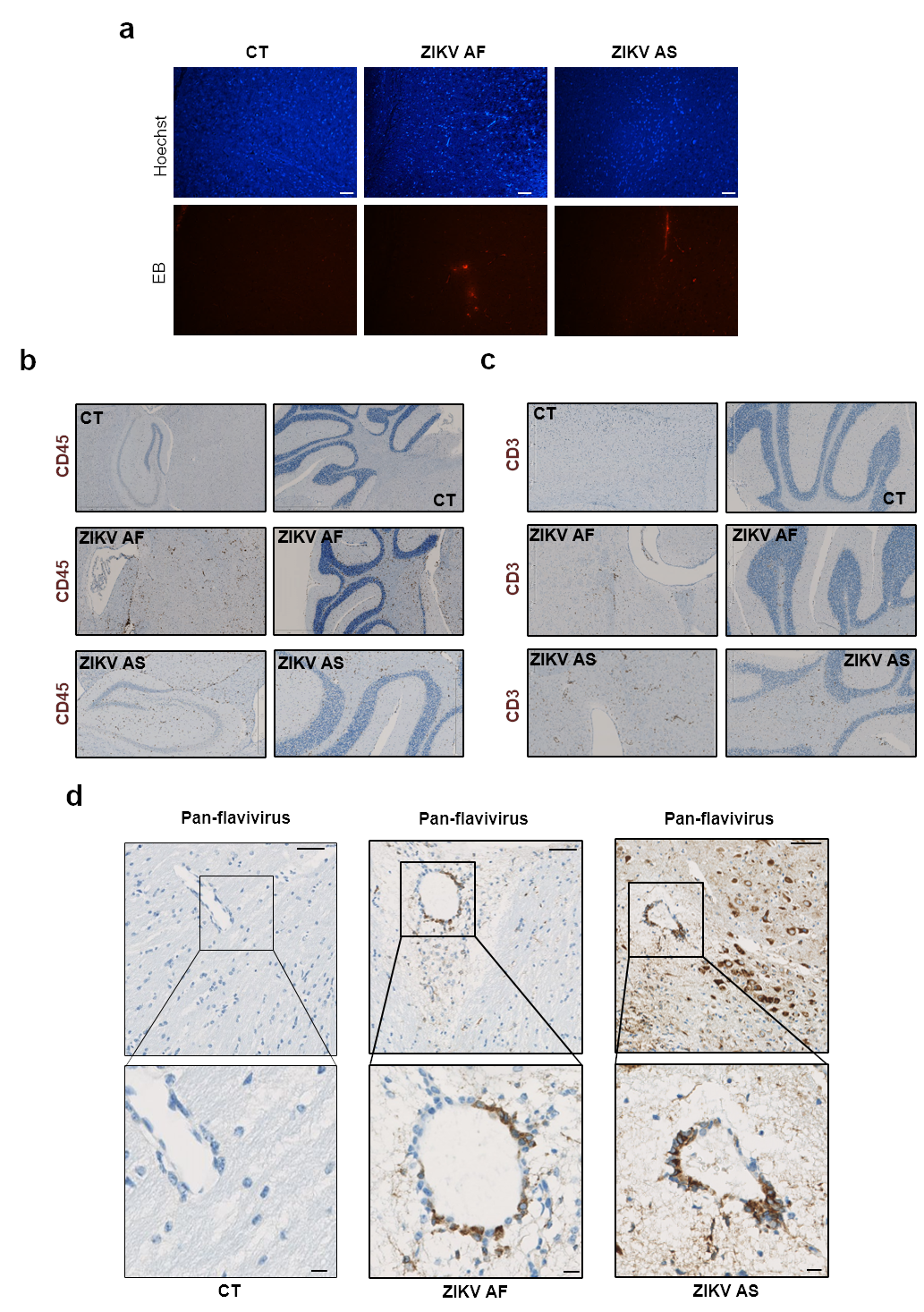

Supplement: FIG S7 [file mBio.01183-20-sf007.tif]

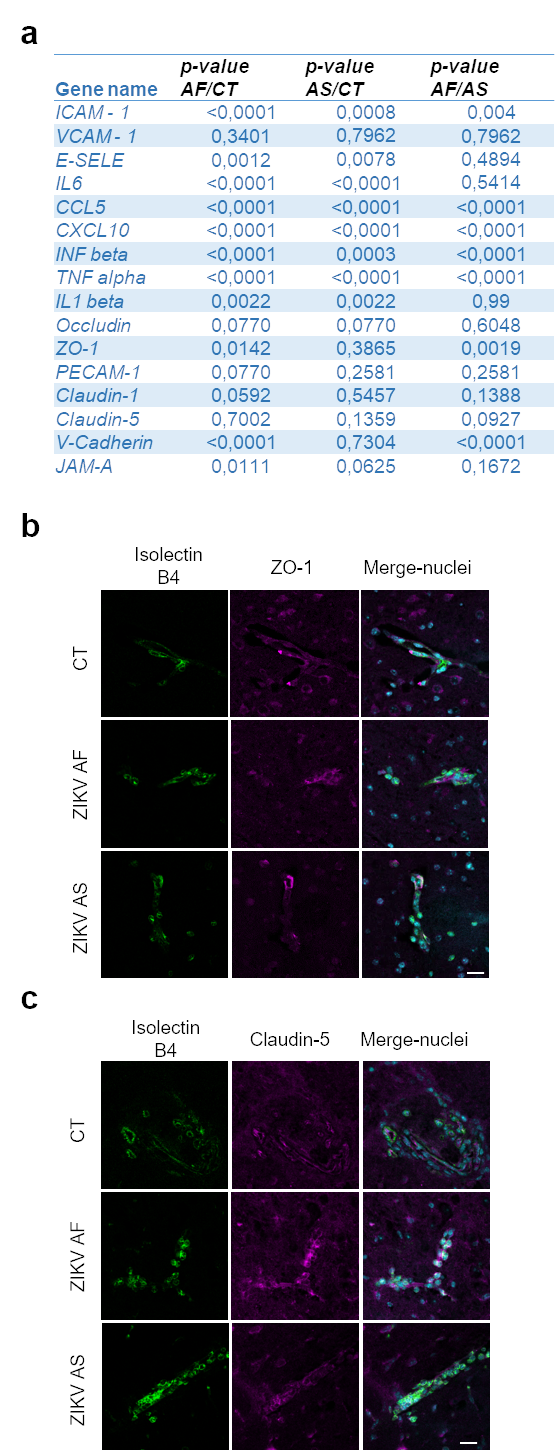

Supplement: FIG S8 [file mBio.01183-20-sf008.tif]
